# Liver Lipidomics, Histology, Transcriptomics, and Clinical Chemistry of Rats Intraperitoneally Treated with Fumonisin B1 for 5 days

**DOI:** 10.1021/acs.jafc.5c05715

**Published:** 2025-09-17

**Authors:** András Szabó, Mária Péter, Gábor Balogh, Zsolt Török, Zoltán Kóta, László Vígh, Omeralfaroug Ali, Botond Timár, György Kövér, Tibor Nagy, Ferenc Olasz, Brigitta Bóta, Örs Petneházy, Patrik Gömbös, Edward Agyarko, Nguyen Anh Thi, Éva Varga-Visi, Melinda Kovács

**Affiliations:** † HUN-REN Biological Research Center, Institute of Biochemistry, Szeged 6726, Hungary; ‡ Single Cell Omics Advanced Core Facility, Hungarian Centre of Excellence for Molecular Medicine, Szeged 6726, Hungary; § Agribiotechnology and Precision Breeding for Food Security National Laboratory, Department of Physiology and Animal Health, Institute of Physiology and Nutrition, Hungarian University of Agriculture and Life Sciences, Kaposvár 7400, Hungary; ∥ Department of Pathology and Experimental Cancer Research, Semmelweis University, Budapest 1085, Hungary; ⊥ Department of Animal Science, Institute of Animal Breeding Sciences, Hungarian University of Agricultural and Life Sciences, Kaposvár 7400, Hungary; # Agribiotechnology and Precision Breeding for Food Security National Laboratory, Institute of Genetics and Biotechnology, Hungarian University of Agriculture and Life Sciences, Gödöllő 2100, Hungary; ¶ HUN-REN-MATE Mycotoxins in the Food Chain Research Group, Kaposvár 7400, Hungary

**Keywords:** fumonisin B1, rat, liver, lipidomics, arachidonic acid, transcriptomics, inflammatory signaling

## Abstract

Fumonisin B1 was intraperitoneally administered at 2.1 μg/animal/day (1×) and 5 and 10 times above (5× and 10×) to male rats for 5 days (*n* = 8/group, *n* = 32). Bodyweight gain decreased in all FB1-treated groups after 3 days, while bodyweight decreased (10×) after 5 days. Feed intake and liver weight decreased (5×, 10×). Clinical chemistry indicated increased total protein, albumin, cholesterol, AST, ALT, and gamma-GT (10×). Histopathology revealed apoptosis, mitosis, hydropic change, mild steatosis, and pericentral glycogen depletion (10×). Shotgun lipidomics showed an increase in sphingosine at 10×, with a dose-dependent depletion of longer-chain ceramide and sphingomyelin molecular species. GM3 ganglioside and free and esterified cholesterol increased linearly with FB1 dose. Arachidonic acid (AA)-containing species of ether-type phosphatidylcholine and phosphatidylethanolamine (PE) displayed a linear dose response; those in diacyl PE and phosphatidylinositol followed logarithmic models. Transcriptomics revealed downregulation of steroid metabolism, while the NF-kB, sphingolipid, PI3K-Akt-PTEN, and TNF signaling pathways were upregulated, critically beyond 5×.

## Graphical abstract

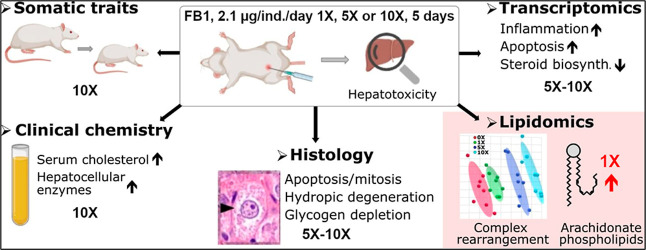

## 1 Introduction

**Occurrence**: fumonisin mycotoxins are mostly produced by filamentous fungi *Fusarium verticillioides* and *Fusarium proliferatum*, posing a global health concern,^1^ and were first isolated and characterized by Gelderblom et al. (1988).^2^ While multiple conformational fumonisin analogues are known (A, B, C, D, P, Py, and La),^3^ the toxicological importance of the B series (ca. 70–75% of total FBs), in particular that of FB1, is critical. The molecule itself is a linear amino pentahydroxy eicosane chain, with two hydroxyl groups esterifying each a tricarballylic acid, being charged and water-soluble.

**Absorption and Excretion**: the intestinal absorption of FB1 in monogastric animals is very low (oral bioavailability is ca. 1–6%;^4^ 3.5% in Wistar rats^5^), and the elimination is quick, partially, but dose-dependently via the urine (monkeys;^6^ rats^7^) and mostly via the feces. The harmful effect of FB1 is species-^8^ and organ-specific,^9^ and typically, liver, lungs, kidneys, heart, brain, and intestines are affected.^10^ Specifically in rats, FB1 target organ is the kidney,^11–13^ but the liver (organ weight and function, as well as hepatocellular integrity) is also compromised.^14,15^

**Accumulation**: fumonisin persistence/accumulation in the liver is interestingly very low and only temporary. Martinez-Larranaga et al. (1999)^5^ reported a ca. twofold accumulation rate, as compared to the plasma (after 10 mg/kg per os dietary dose) in Wistar rats. A possible explanation of this is that FB1 clearance is quite quick (∼3 days in chickens^16,17^ and in pigs,^18^ ∼4 days in rats^19^) but it is noteworthy that FB1 shows biliary excretion and enterohepatic circulation (in pigs^18^) and intestinal (partial or full), but not hepatic hydrolysis.^20^ Once the host animal is exposed to repeated FB1 intake (chickens^16,17^ for 4–9 days, turkeys^21^ for 14 days, and ducks^22^ for 12 days), or even to a single dose (rabbits;^23^ vervet monkeys;^20^ rats^24^), a characteristic hepatotoxic effect is exerted.

**On the organ level**, in rats, decreased liver weight was reported (10 days, 20–50–100 mg/kg,^14^ but not proven after 5 days at a high level, 50 mg/kg^25^) in short studies. Additionally, dose-dependence for the decreasing liver weight in rats in a high-exposure test (99, 163, 234, and 484 mg/kg for 4 weeks) was reported.^26^ The no observed adverse effect level (NOAEL) for subchronic studies has been estimated to be relatively high for rat liver. Voss et al. (2001) reported the NOAEL for Sprague–Dawley rats to be between 50 and 150 and for Fischer 344 rats it is over 81 mg/kg feed in males, based on histopathology and biochemical endpoints.^8^ In contrast, the lowest NOAEL for rats in subchronic studies was set to 0.2 mg/kg BW/day, being equivalent to a dietary dose of ca. 1.6 mg/kg diet.^27^

**At the cellular level**, liver histopathology generally reveals shrunk, round-shaped cells and typical cell detachment.^8^ According to the study, inflammation is absent, and meanwhile cell shape is apoptotic (condensed chromatin), the process appears to be single-cell oncotic necrosis,^8^ while the presence of apoptosis was also proven.^26^ In a 10-day setting, we additionally reported solitary hepatocellular apoptosis and the formation of Councilman bodies, along with vacuolar hepatocellular degeneration in male Wistar rats (at a 100 mg/kg dose, 6 times higher than the present 10× dose).^14^

**On a biochemical level**, the hepatotoxic effect is met by the characteristic mode of action of FB1, the inhibition of ceramide synthesis,^28^ affecting all 6 known CerS (ceramide synthase) isoforms (in piglets^9^). According to Riley and Voss (2006), CerS inhibition leads to cellular free sphinganine (Sa) (and to a lesser extent sphingosine^29^ (So)) accumulation.^30^ Cells react to this toxic stimulus with augmented phosphorylation of Sa and So (to Sa-1-P and So-1-P) and subsequent hydrolysis (to phosphoethanolamine and a fatty aldehyde).^30^ These effects result in increasing So, Sa, and their phosphorylated bases’ concentrations and in dropping Cer and upstream sphingolipid synthesis.

In addition, CerS1-6 provide organ-specific distribution^9^ and have characteristic and selective preference toward long-chain fatty acyl-CoA species (e.g.: CerS5 for C16; CerS2 for C22–24 ^31^). The interaction of FB1 and the CerS enzyme happens at two binding sites: the aminopentol part binds to the sphinganine-specific site (inducing sphingolipid metabolism disturbance), and the tricarballylic acid side chains interact with the fatty acyl-CoA binding site.^32,33^ Consequently, FA synthesis or metabolism substrates become perturbed; FB1 exerts a further disturbing effect on the FA profile via the induction of oxidative stress, as reported at high doses over 50 mg FB1/kg diet in male Wistar rats.^14^ Moreover, FB1 does not selectively perturb sphingolipid metabolism but also affects the metabolism of other, e.g. fatty acid (FA)-containing lipid fractions (P-choline, P-inositol, and PE, (rat kidney after 20–50–100 mg/kg FB1 for 5 and 10 days^15^), glycerophospholipidome of the pig liver and lung^35^). Further consequent changes include polyunsaturated FA (C18–C20, *n*6 and *n*3) liberation from the membrane lipids entering the cyclo- or lipoxygenase, and P450 enzyme mediated oxylipin formation, only after 4–9 days; hepatic changes in the cited study were not related to any inflammatory process in chickens.^36^

Our multimodal study had the hypothesis that subclinical intraperitoneal exposure to FB1 (and its multiplications) affects the hepatocellular membrane integrity partly by disarranging the lipidome. We applied classical (clinical chemistry and histology) and high-content (transcriptomics and lipidomics) analytical techniques to test the hypotheses: (i) if early signs of hepatotoxicity in rats emerge after only 5 days; (ii) whether we can identify FB1-specific, new (lipid) biomarkers and metabolic and genetic pathways; (iii) whether the adverse effects exhibit a dose-dependent response within the 0–1×–5×–10× dose range; and (iv) whether we find associations between the different-level cellular features to explain the mechanism of FB1 mode of action.

## 2 Materials and Methods

### 2.1 Experimental Animals, Keeping, and Feeding

Altogether 32 HAN/Wist SPF adult male rats (total population mean initial body weight: 284.5 ± 17.5 g) were enrolled in the study. During acclimatization (7 days) and the subsequent mycotoxin treatment period (5 days), paired rats were allocated into Eurostandard IV-type polycarbonate cages. Feed and drinking water were offered *ad libitum*. The light program was 12:12 h light–dark (automatically adjusted). The feed was ssniff R/M-Z + H (ssniff Spezialdiäten GmbH, Soest, Germany). Feed consumption and body weight were recorded daily. The 32 rats were allocated into 4 equal groups of 8 individuals as follows: control (no mycotoxin administration, “C”), 1×, 5× and 10× (2.1, 10.5, and 21 μg fumonisin B_1_/individual/day, respectively).

### 2.2 FB1 Administration Protocol

FB1 administration was performed via intraperitoneal (i.p.) injections, in a total of 5 times, daily once, each day at 8 a.m., when the bodyweight was also measured. The injected volume was 0.5 mL/individual/day; C received sterile physiological saline. The fumonisin B1 groups’ stock solutions (FB1 toxin (Fumizol Ltd., Szeged, Hungary, purity ≥ 99%, dissolved in physiological saline) were prepared at the concentrations of 4.2, 21, and 42 μg/mL, and each time 0.5 mL of these were injected into the animals. A recovery check of FB1 from the stock solution was performed daily with a Shimadzu 2020 LC/MS system (Shimadzu, Kyoto, Japan). The accuracy of the results was within ±0.2%. FB1 level was set to 0.2 mg/kg bodyweight/day, which is the lowest NOAEL for rats in subchronic studies.^27^ This dose is equivalent to a dietary FB1 dose of 1.6 mg/kg of diet. The i.p.-administered toxin equivalent amounts were calculated based on ∼3.5% intestinal absorption rate when applying oral dosage (Martinez-Larranaga et al. 1999).^5^

### 2.3 Slaughter, Sampling, and Ethical Allowance

After 5 days of FB1 treatment, on the next day at 8 a.m., rats were anesthetized with diethyl ether, and after taking blood from the retro-orbital plexus (into K_2_EDTA tubes, Vacutainer, Oakville, ON, Canada), cervical dislocation was performed, and animals were exsanguinated. Rats were immediately dissected, and liver tissue samples were collected into cryovials immediately immersed into liquid N_2_. After snap freezing, samples were stored at −80 °C. The experimental protocol was authorized by the Food Chain Safety and Animal Health Directorate of the Somogy County Agricultural Office, under the permission number SO/31/01287-3/2022 (KA-3621).

### 2.4 Histological Sample Preparation

Liver samples were stored in 10% neutral buffered formalin and were embedded into paraffin. For light microscopic analysis, microtome slides of 5 μm were prepared and stained with hematoxylin–eosin and periodic acid-Schiff (PAS) staining (not digested procedure), respectively. All stained slides involved in this study were subjected to whole slide digitalization using a Pannoramic 1000 Scanner (3DHistech Ltd., Budapest), and their visual analysis and scoring were done within the SlideViewer program (3DHistech). The main pathological alterations have been described and scored according to their extent and severity as follows: 0 = no change, 1 = minimal, 2 = mild, 3 = moderate, 4 = severe. Total score value was defined as the sum of all scores in all identified histological changes per sample.

### 2.5 Shotgun Lipidomics

The tissue samples were weighed, cut into max. 200–300 mg pieces, and homogenized in a calculated volume of water with a bullet blender homogenizer (Bullet Blender Gold, Next Advance, Inc., Averill Park, NY, USA) in the presence of zirconium oxide beads (0.5 and 1 mm), at a speed level of 8 for 2 × 3 min at 4 °C to obtain a watery homogenate with close-to-identical concentrations for all samples.

A portion of the homogenate (10 μL, corresponding to ca. 2–3 mg wet weight) was subjected immediately to a one-phase methanolic lipid extraction, which ensures good (∼95% or higher) recovery for various lipid classes, as shown previously.^37^ The tissue homogenate aliquot was sonicated in 0.5 mL of methanol containing 6 μg of PC(40:0) and 4 μg of d7-Chol (as extraction standards) and 0.001% butylated hydroxytoluene (as an antioxidant) in a bath sonicator for 5 min, then shaken for 5 min and centrifuged at 10,000*g* for 5 min. The supernatant was transferred to a new Eppendorf tube and stored at – 20 °C until mass spectrometry (MS) analysis.

Electrospray ionization (ESI)-MS analyses were performed on a high-sensitivity, high-resolution Orbitrap Fusion Lumos instrument (Thermo Fisher Scientific, Bremen, Germany) equipped with a TriVersa NanoMate robotic nanoflow ion source (TriVersa NanoMate, Advion BioSciences, Ithaca, NY, USA), using chips with spraying nozzles of 5.5 μm diameter. The instrument was fully calibrated before analysis. The ion source was controlled by Chipsoft v8.3.1 software. The ionization voltages were +1.3 and −1.9 kV in positive and negative modes, respectively, and the back-pressure was set to 1 psi in both modes. The temperature of the ion transfer capillary was 260 °C. Acquisitions were performed at the mass resolution *R*_*m*/*z*=200_ = 240,000, and signals were recorded in the centroid mode.

Based on preliminary injections, 3 μL of extract was further diluted with 120 μL of infusion solvent mixture (chloroform/methanol/iso-propanol 1:2:1, by vol.). The infusion solvent was spiked with an internal standard mix (Avanti Polar Lipids, Alabaster, AL, USA; Appendix Table_S13). Next, the mixture was halved, and 5% dimethylformamide (additive for the negative ion mode) or 3 mM ammonium chloride (additive for the positive ion mode) was added to the split sample halves. 10 μL of the solution was infused, and data were acquired for 2 min.

For free cholesterol determination, 100 μL of methanolic extract was evaporated to dryness, and 180 μL of chloroform/acetyl chloride = 5:1 mixture was added to the residue to form the acetate derivative of free sterols. The reaction mixture was left for 1 h at room temperature, evaporated to dryness, and reconstituted in 75 μL chloroform/methanol = 1:1. For MS analysis, 3 μL of the derivative was diluted with 60 μL of infusion solvent (chloroform/methanol/isopropanol = 1:2:1, by vol.) in the presence of 100 mM sodium carbonate. Cholesterol acetate (CholAc) was detected in the positive ion mode at +1.5 kV ionization voltage at the mass resolution *R*_*m*/*z*=200_ = 240,000.

Phosphatidylcholine (diacyl, PC and alkyl-acyl, PC-O), phosphatidylethanolamine (diacyl, PE and alkenyl-acyl, PE-P), phosphatidylinositol (PI), phosphatidylserine (PS), phosphatidic acid (PA), phosphatidylglycerol (PG)/bis­(monoacylglycero)­phosphate, cardiolipin (CL), and the lyso derivatives LPC, LPE, LPI, LPS, LPG, and LCL as well as ceramide (Cer), sphingosine-1-phosphate (So-1-P), GM3 ganglioside, and free fatty acids (FFA) were detected and quantified using the negative ion mode, whereas sphingosine (SPH(18:1:2) or So), sphinganine (SPH(18:0:2) or Sa), sphingomyelin (SM), diacylglycerol (DG), triacylglycerol (TG), cholesteryl ester (CE), and free cholesterol (Chol) were detected and quantified using the positive ion mode.

Raw MS spectra were converted to platform-independent mzML files, and lipid species were identified with the LipidXplorer software.^38^ Identification was made by matching the *m*/*z* values of their monoisotopic peaks to the corresponding elemental composition constraints. The mass tolerance was set to 3 ppm. Data files generated by LipidXplorer queries were further processed by self-developed Microsoft Excel macros. For quantification, in most cases, we averaged 50 MS1 scans, and then we integrated signal intensities after built-in C13 isotopic correction (both types I and II). Relative analyte concentrations were determined from the concentrations of matching internal standards (Table) added after extraction. For Sa-1-P and So-1-P, we used MS1 in SIM mode to increase the detection sensitivity, while sphingoid bases were quantified from MS3 fragments to avoid overlapping background signals present in MS1 survey scans. Calculation of recovery for PC(40:0) afforded good results (ca. 95%, Appendix Table_S13) and revealed no difference between the four experimental animal groups. Absolute concentrations were calculated based on the amount of the extraction standards.

Lipid species were annotated with sum formulas according to the shorthand notation for lipid structures.^39^ For glycero­(phospho)­lipids, e.g., PC(38:4), the total numbers of carbons followed by double bonds for all chains are indicated. For sphingolipids, the sum formula like SM(34:1:2) specifies first the total number of carbons in the long chain base and FA moiety and then the sum of double bonds in the long chain base and the FA moiety, followed by the sum of hydroxyl groups in the long chain base and the FA moiety. We note that the sum formula of glycerophospholipids might describe different fatty acyl combination isomers. To resolve this, data-dependent tandem MS2 or MS3 fragmentation experiments were performed based on mass lists from survey scans.^40^ Similarly, PC/PE-O­(*x*/*y*) (alkyl-acyl) and PC/PE-P­(*x*/*y* – 1) (alkenyl-acyl) species, as well as PG and BMP species, are also isomeric. These types of isomerisms were not resolved in the current work; therefore, the coexisting isomers (if any) were quantified collectively.

The fatty acid profile of the rat feed (Appendix Table_S06) was analyzed with gas chromatography and a flame ionization detector.^41^

### 2.6 Transcriptomics

Tissue specimens were directly sampled into TriReagent (Merck T9424), mildly sedimented, and snap frozen in liquid nitrogen. mRNA expression profiles of 8 liver samples per group were assessed using the Agilent GENEXP-4× SurePrint G3 Rat Gene Expression package (Agilent Technologies, California, USA) following the manufacturer’s instructions. RNA extraction was done with the Trizol reagent (Amresco, USA), and mRNA extraction results are provided in the Appendix Table_S12. The cRNA was hybridized onto the microarray slides, which were scanned on an Agilent G2505C Microarray Scanner using Agilent Scan Control A.8.5.1 software, and the fluorescence signal was extracted using Agilent Feature Extraction software v10.10.1.1 with default parameters. Microarray data and experimental details are accessible through GEO Series accession number: https://www.ncbi.nlm.nih.gov/geo/query/acc.cgi?acc=GSE286344.

### 2.7 Clinical Chemistry

A Beckman Coulter AU5811 automated equipment (Beckman Coulter, Inc., Brea, CA, USA) was used for the measurements with respective diagnostic kits (see Appendix Table_S13), using plasma samples after frozen storage at −80 °C.

### 2.8 Statistical Analysis

Somatic results were evaluated with ANOVA and Tukey post hoc test, to detect intergroup differences. The grouping variable was the mycotoxin dose (d*f* = 3). Linear and logarithmic regressions were used to test dose response; results are only interpreted if the model fitting gave an *R* squared value over 0.6; this fitting was tested exclusively in cases where ANOVA provided significant intergroup differences. In the statistical analyses, differences between groups were considered significant when *P* values were <0.05.

The histopathological evaluation was ranked from 0 to 4, according to the severity of the changes. The continuous lipidomics and the ranked histopathology data were tested for correlation with the Spearman’s rho rank correlation method, while clinico-chemical data correlations were analyzed with Pearson’s method. All above analyses were performed using IBM SPSS 29.0 (2023) software.^42^

Multivariate analysis of lipidomic data was performed with R project version 4.1.2 (2017)^43^ and the mixOmics package (6.18.1.).^44^ KEGG pathway and gene mapping were performed with the Pathview application.^45^ Partial Least Squares Classification (Discriminant Analysis, PLS-DA) was performed for dimension reduction and variable selection for classification with the highest accuracy^46^ on the MetaboAnalyst GUI.^47^

In transcriptomics, to evaluate samples and to identify possible outliers, we used unsupervised clustering of the normalized data using the “pheatmap” package. Gene ontology (GO) enrichment analysis was performed using the “clusterProfiler” package (version 4.10.1) (Wu et al., 2021).^101^ Pearson correlations were calculated for the lipidomics and transcriptomics data inter-relationship. Both analyses were done in R.^43^

## 3 Results

### 3.1 Somatic Traits (FB1 Dose and Exposure Time Dependence)

Final bodyweight (BW) was significantly lowered in the 10× group (vs *C*, Figure 1A). The daily BW gain was lower in the 5× and 10× groups from day 3 (vs *C*, Figure 1B); BW loss was recorded in 10× only. The daily feed intake (FI) decreased in all of the FB1 groups (Figure 1C). We recorded FB1 dose-dependence for the total BW change, the cumulative feed intake (FI), and the ex vivo liver weight (Figure 1D and Appendix Table_S02).

**Figure 1:**
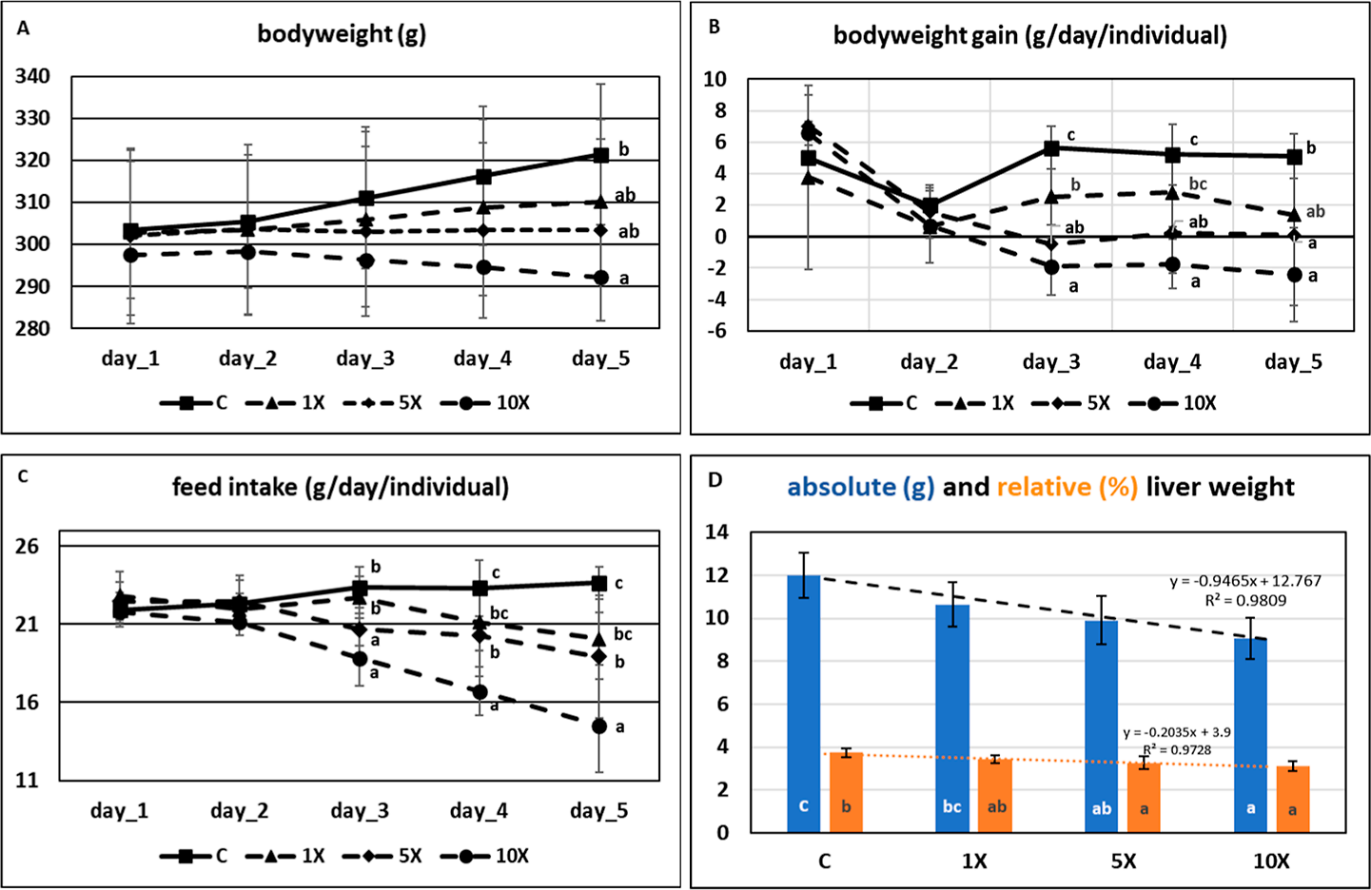
(A) Bodyweight of the rats during the 5 study days; (B) bodyweight gain during the 5 day period in the 4 groups; (C) feed intake during the FB1 administration period; (D) absolute and relative liver weight at the end of the experiment. (dots and columns represent means ± SD) (a–c in the plots indicate significant intergroup differences (*p* < 0.05) of means at the distinct sampling days if the indices differ).

### 3.2 Clinical Chemistry

The full clinico-chemical data set can be found in the Appendix Table_S07. Total protein, albumin, uric acid, and the calculated globulin concentration increased in the 10× treatment. Among lipids, the triglyceride concentration decreased, while the total HDL and LDL cholesterol concentrations increased at the 10× level. Within hepatic enzymes, aspartate aminotransferase (AST), alanine aminotransferase (ALT), gamma-glutamyl-transferase (GGT), and lipase increased in the 10× group. In several cases, the elevations were dose dependent (Figure 2).

**Figure 2:**
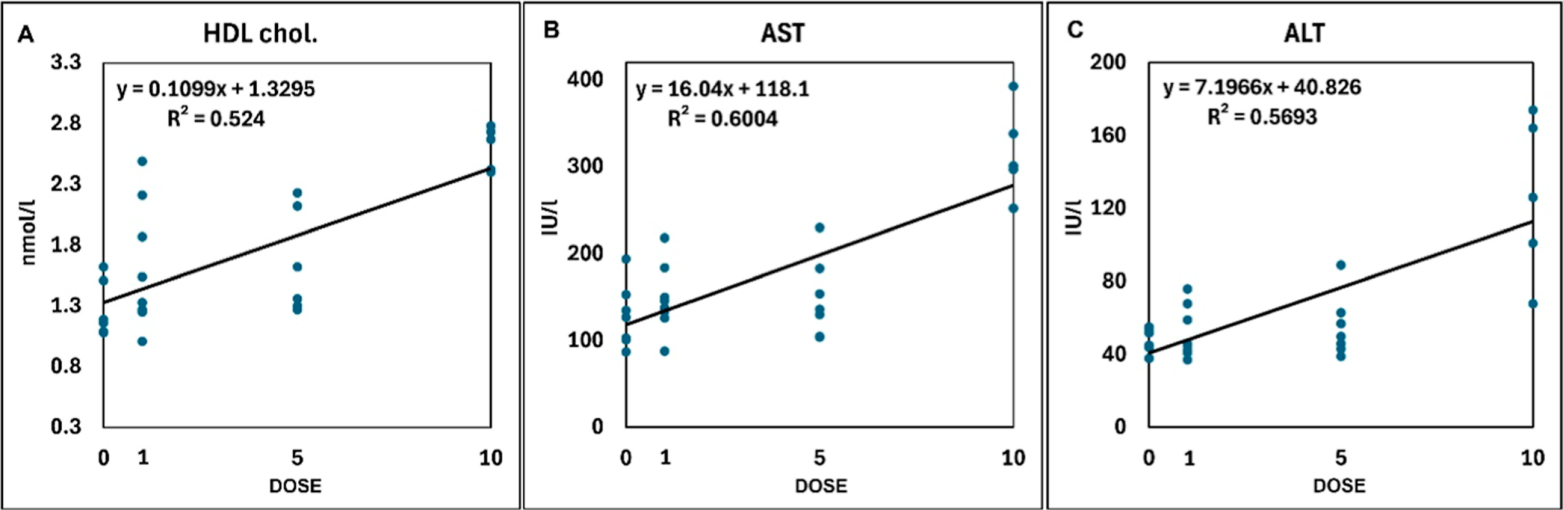
Linear, dose-dependent alterations of the HDL cholesterol concentration (A), AST (B), and ALT (C) activities in the 4 experimental groups after 5 days (dots represent individual values).

### 3.3 Histopathology

To assess FB1-induced liver damage, the following histologic changes were quantified: fatty change (steatosis), hydropic degeneration, apoptosis/mitosis/regenerative signs, hepatocellular hypertrophy, necrosis, portal changes, and loss of PAS positivity (indicative of glycogen depletion). Group-assorted data are summarized in Table 1, whereas individual data are given in the Appendix Table_S08. We identified typical hepatotoxicosis signs at 5× and 10×, such as hydropic degeneration and hepatocellular hypertrophy (Figure 3A). Additionally, apoptosis (Councilman bodies) and regenerative signs (mitosis) were observed simultaneously and were located around the central vein (Figure 3B). Portal changes were also detected as mild, yet apparent, lymphocytic infiltration and inflammation (without ductal modifications) (Figure 3C). PAS staining revealed strictly pericentral color intensity loss on the sections, likely due to glycogen depletion (Figure 3D). Noteworthy, necrosis was fully absent.

**Table 1:** Mean and Range Descriptive Data of the Distinct Histological Findings Logged in the Liver Samples^a^

group	C			1×			5×			10×		
	mean	min.	max.	mean	min.	max.	mean	min.	max.	mean	min.	max.
fatty change	0.25	0	1	0	0	0	0	0	0	0.25	0	1
hydropic degeneration	0.13	0	1	0.13	0	1	0.75	0	1	1.13	1	2
apoptosis/mitosis/regenerative sign	0	0	0	0	0	0	2	1	3	2.75	2	4
hepatocellular hypertrophy	0.38	0	1	0.38	0	1	0.88	0	1	1	1	1
necrosis*	0	0	0	0	0	0	0	0	0	0	0	0
portal changes	0	0	0	0	0	0	0.38	0	1	1	1	1
loss of PAS positivity	0.88	0	1	0.75	0	1	2.25	1	4	2.87	2	4
total score value	1.63	0	3	1.25	0	3	6.25	3	10	9	7	12

a Means are calculated for *n* = 8/group, *: checked but not discovered, and PAS: periodic acid Schiff staining.

**Figure 3:**
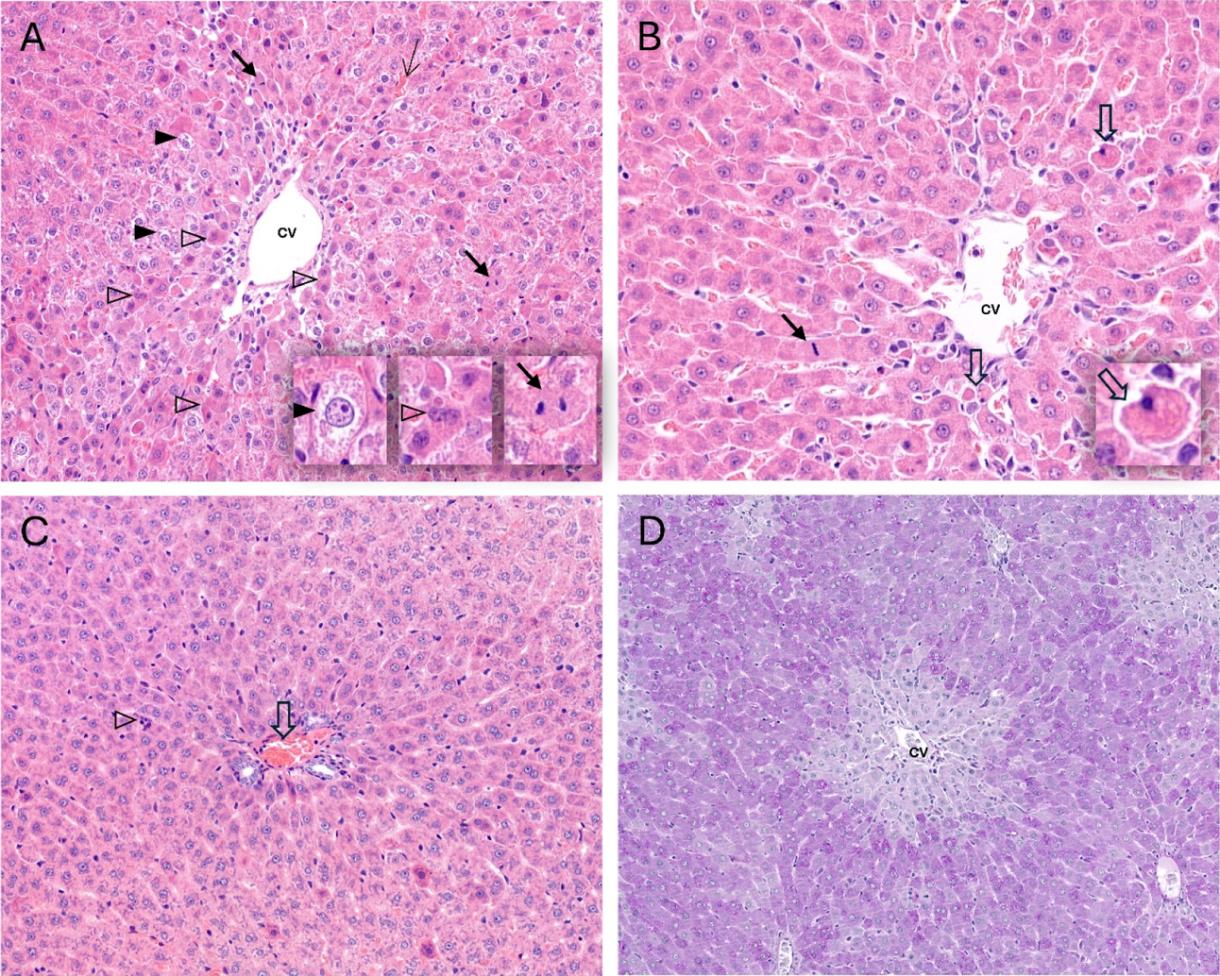
Histologic changes observed in the rat liver at 10× FB1 dose after 5 consecutive days. (A) Hydropic change of hepatocytes (filled arrowhead and insert) with pericentral mitotic figures (arrow and insert) and with parenchymal collapse with atrophic hepatocytes (empty arrowhead and insert) (63×, HE staining). (B) Presence of apoptosis (Councilman bodies, empty arrowhead, insert) and mitotic figures (arrow) in a pericentral (CV: central vein) predominance (63×, HE staining). (C) Minimal portal changes, predominantly congestion (empty arrow), with occasional lymphocytic infiltrates in the periportal region (empty arrowhead) (63×, HE staining). (D) Loss of PAS staining intensity (positivity) in the pericentral region (63×, PAS staining).

### 3.4 Liver Lipidomics

#### 3.4.1 Overview of Lipidomic Data

In our untargeted, shotgun ESI-MS measurements, we identified and quantified ca. 250 lipid species, encompassing 25 lipid classes, including membrane, signaling, and storage lipids (Appendix Table_S03). Partial Least Squares Discriminant Analysis (PLS-DA) revealed clear separation between the control and all treated groups, indicating that the FB1 treatment largely and quickly disarranged the hepatic lipidome (Figure 4A). In addition, the heatmap representation based on 50 lipid molecular species in hierarchical cluster analysis plots the complex lipidomic rearrangement upon increasing FB1 concentrations (Figure 4B). From the large data set first, we interpret changes systematically at the lipid class level (Figure 4C,D, Appendix Table_S03).

**Figure 4:**
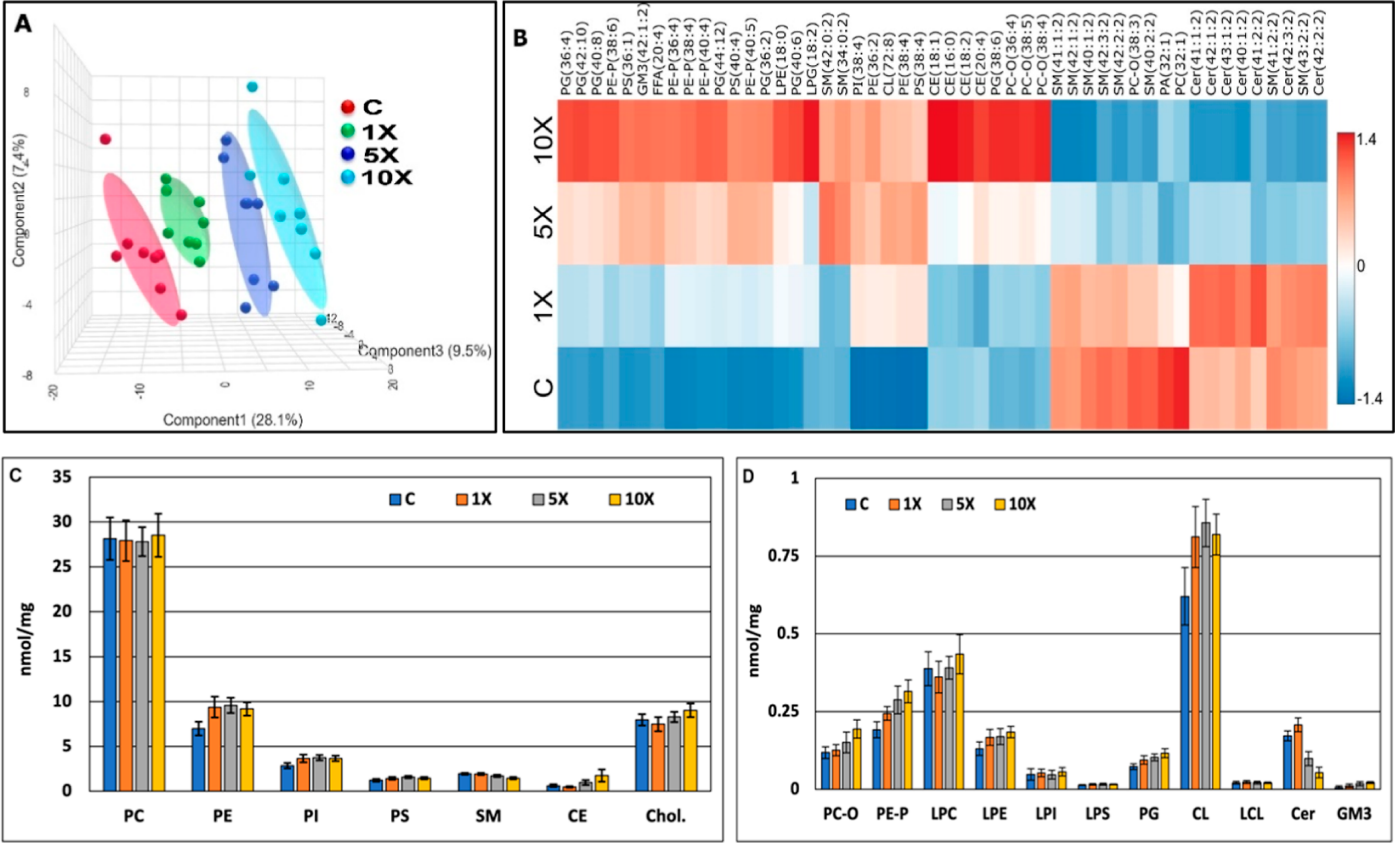
Overview of the lipidomic response to i.p. FB1 treatment. (A) PLS-DA score plot and (B) heatmap representation of hierarchical cluster analysis based on the entire hepatic lipidomics data set (the top 50 most significant species were selected based on ANOVA, Euclidean distance, and the clustering algorithm Ward; only group averages are shown; the heat color code represents normalized values (*z*-scores)), FB1-induced changes at the lipid class level in the (C) major and (D) minor lipid classes (bars represent means of 8 ind. values ± SD).

To evaluate the dose response on the FB1 treatment, the lipid class fold changes and linear or logarithmic fittings were calculated (Appendix Table_S03 and Appendix Table_S04). Results indicate a slight, but dose-dependent concentration increase of plasmanyl PC (PC-O), while marked concentration increase for PE-P, PG, and LPG, GM3 ganglioside, and cholesteryl esters (CE). Dose-dependent depletion was found for total ceramides (Cer) and SM.

#### 3.4.2 Sphingolipids and Ceramide Synthases

First, we examined the alterations of the hepatic sphingoid bases and sphingolipids because, due to its structural similarity to sphinganine and FA-CoA, FB1 is known to inhibit ceramide synthases (CerS). In our experimental setup, we detected significant reduction in the expression of CerS2, CerS3, and CerS4 (which produce longer chain ceramide (Cer) species) at the 10× level, while CerS1 expression increased in the 5× and 10× groups (Figure 5A); CerS5 and 6 were nonresponsive on FB1.

**Figure 5:**
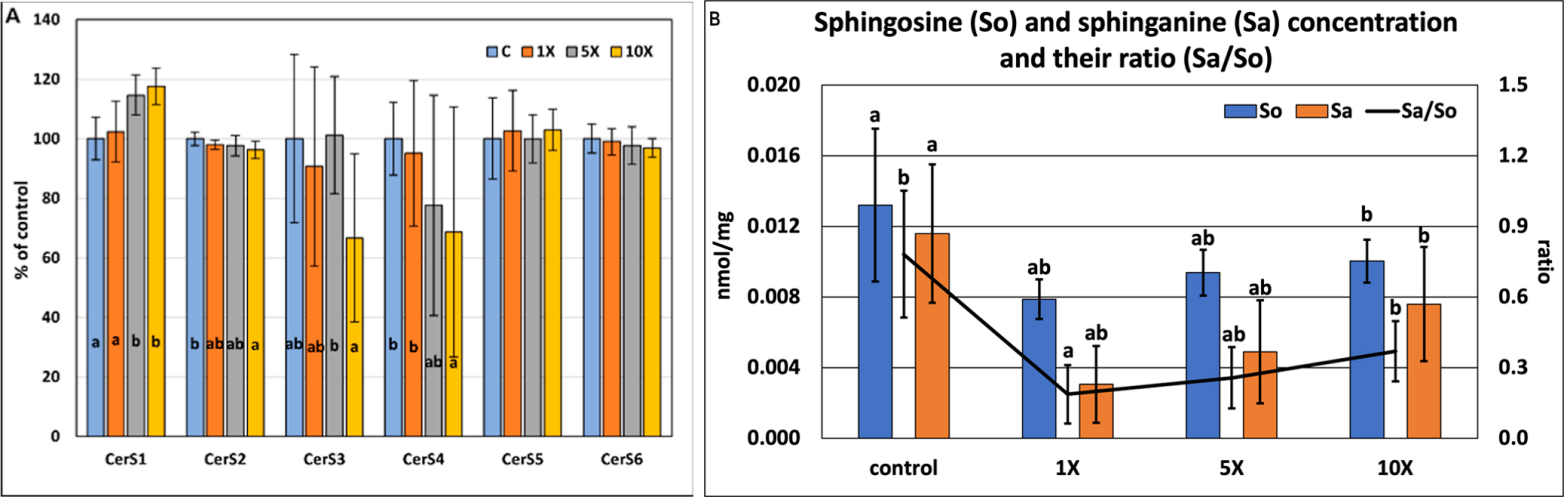
(A) CerS isoforms’ relative expression in the rat livers (100% refers to the control). (B) Concentration of hepatic sphinganine (Sa) and sphingosine (So), and their ratio. (a,b: different indices mark significantly different group means at *p* < 0.05 by ANOVA).

We observed a slight increase in the concentration of sphingosine (So), a drop in that of sphinganine at 1× and an increase beyond, and the Sa/So ratio followed this pattern (Figure 5B); the concentrations of phosphorylated derivatives of Sa (and So) remained below the limit of detection in all four groups. We detected a significant decrease in the overall Cer level due to the higher-dose FB1 treatments, while the 1× treatment resulted in significant Cer elevation due to an increase in all major Cer molecular species, e.g., Cer(42:1:2) (Figure 6A and Appendix Table_S03). Sphingomyelin (SM), the major membrane-forming sphingolipid, displayed no change in response to the 1× treatment but showed significant reductions at higher FB1 dosages at a lipid class level (Appendix Table_S03 and Appendix Table_S04). Behind this overall decrease, at a lipid species level, we observed remarkably reduced concentrations for the abundant longer-chain species (*C* > 40), like SM(42:2:2) or SM(42:1:2), while the abundant shorter-chain SM(34:1:2) provided increasing concentrations (Figure 6B). Interestingly, the amount of the low-abundance glycosphingolipid ganglioside GM3(42:1:2) elevated with the increasing FB1 dose (Figure 6C). Noteworthy, most of the depicted sphingolipid molecular species provided linear dose-dependent concentration decreases with good linearity; e.g., the coefficient of determination was *R* ^2^ = 0.82 and *R* ^2^ = 0.79 for Cer(42:1:2) and SM(42:1:2), respectively (Appendix Table_S03).

**Figure 6:**
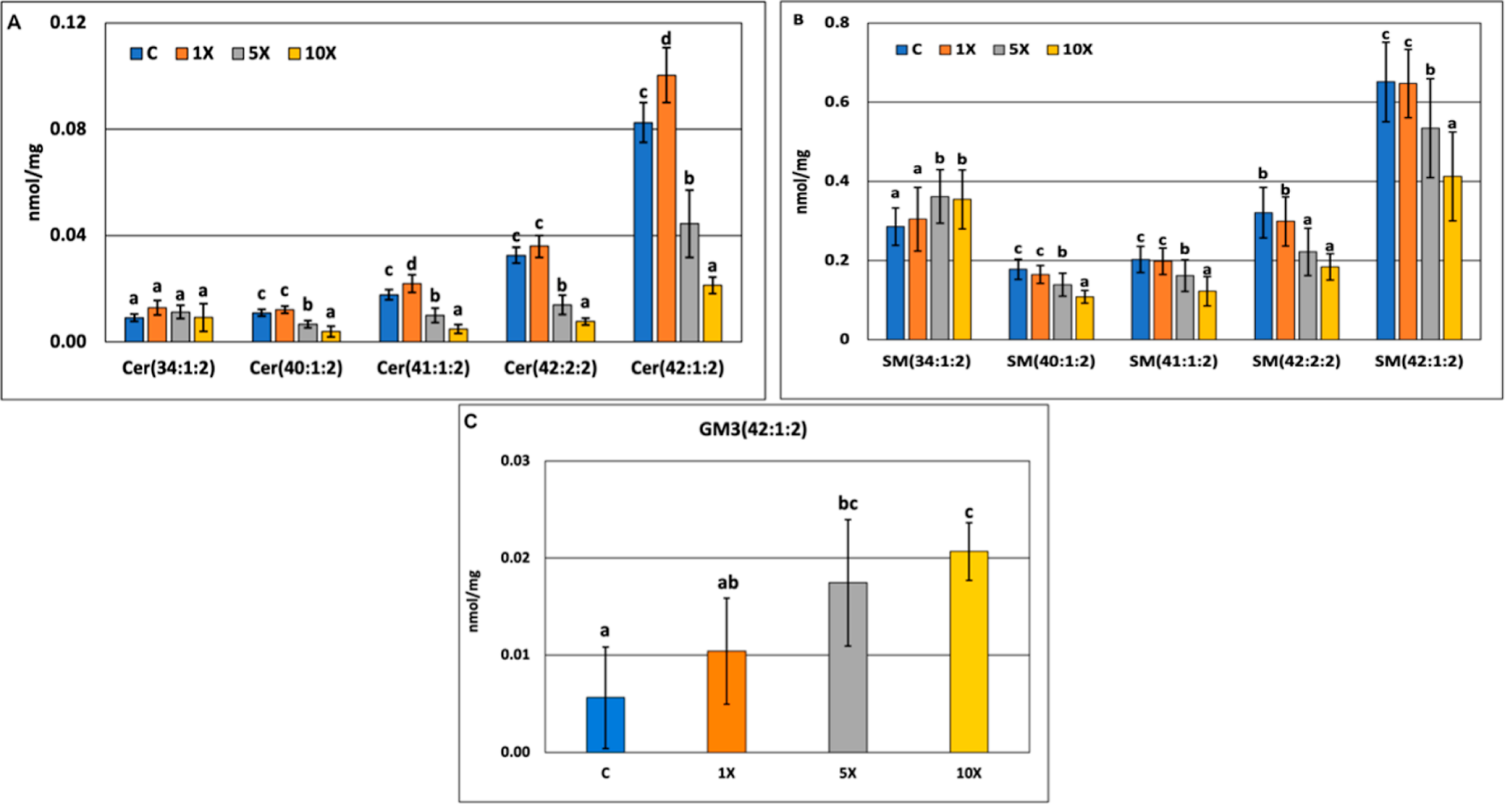
Dose-dependent alterations of some Cer (A), respective SM (B), and the single identified GM3 ganglioside (C) molecular species. (a–c: different indices mark significantly different group means at *p* < 0.05).

#### 3.4.3 FB1 Impacts Glycerophospholipid Metabolism

Due to the interconnection of SL degradation and PE synthesis pathways, combined with the modulatory effect of FB1 on fatty acid metabolism, FB1 might exert a deeper impact on the whole hepatic lipidome beyond the disturbance of SL metabolism. Grouping glycerophospholipid (GPL) concentration data according to lipid classes showed different responses. At the lipid class level, diacyl phosphatidylcholine (PC), the most abundant membrane lipid, did not alter, whereas the other major structural lipids, PE, PI, and PS, increased as a result of FB1 treatment (Figure 4C and Appendix Table_S03). As a consequence, the PE/(PC + PI) ratio increased significantly (Appendix Table_S11). Several minor GPL classes, the ether lipids PC-O and PE-P, PS, PG, CL, and the lysolipids LPE and LPG, were elevated as well. At the lipid species level, we calculated the average phospholipid species profile for the sum of major membrane lipids (PC, PE, PI, and PS; Appendix Table_S03). This revealed that the most remarkable changes occurred in the most abundant GPL(38:4) species, in which stearic acid (18:0) and arachidonic acid (20:4, AA) esterify the *sn*1 and *sn*2 positions on the glycerol backbone, respectively. In addition, based on a more detailed analysis at the lipid species level, we realized that the enriched lipids displayed two different response types (Figure 7). We illustrate this phenomenon with examples of selected lipid species. Linear dose response was detected, e.g., for the AA-containing ether lipid species PC-O(38:4) and PE-P(38:4), whereas logarithmic models provided good fits to describe the increase in the abundant PE(38:4), PI(38:4), and PS(38:4). Importantly, the logarithmic elevation type in AA-containing species was the largest that occurred during the lipidome rearrangement due to FB1, accounting for ca. 35% of all positive changes in the 1× group compared with the control. We also note that a similar dose-dependent trend was observed in the major tetra-linoleyl CL species, CL(72:8) (Figure 7).

**Figure 7:**
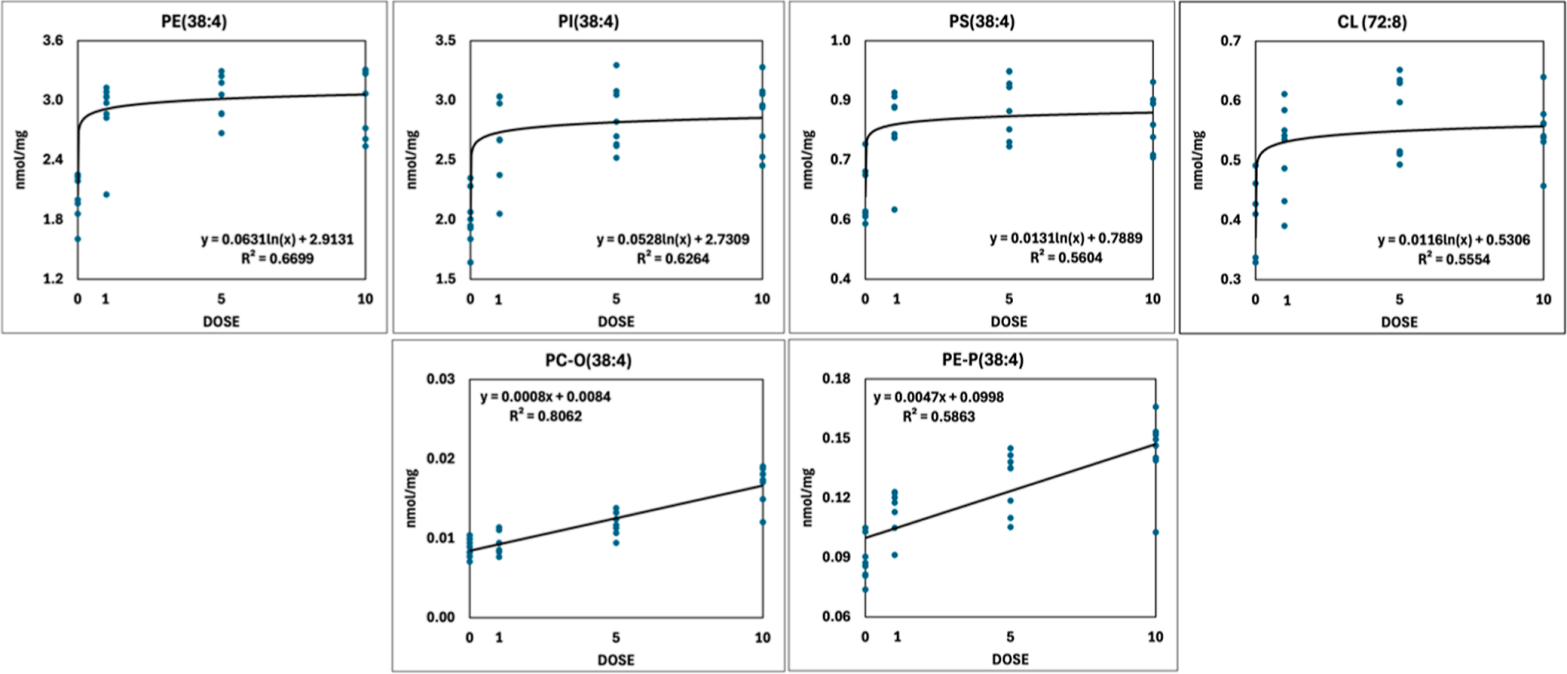
FB1-induced logarithmic and linear dose–response curves of the AA and linoleic acid (CL(72:8) (tetra-linoleoyl CL)) containing lipid molecular species of the glycerophospholipidome (dots represent individual values, *n* = 32).

#### 3.4.4 Neutral Lipid Lipidomics

Among neutral lipids, cholesterol increased significantly (*C* vs 10×) (Figure 4C and Appendix Table_S03). Its esterified form, the storage lipid cholesteryl ester (CE), provided a good linear FB1 dose response, especially the major, AA-containing species CE(20:4) (Figure 8). Triglyceride (TG), the other storage lipid, altered in a species-dependent manner, but not at the class level. The concentration of shorter-chain (*C* ≤ 50), less unsaturated TG species decreased, while the level of longer-chain (*C* > 58), highly unsaturated species increased with increasing mycotoxin concentration (Appendix Table_S03). Among the elevating components, we identified several AA-containing species, e.g., TG(58:10) (Figure 8). In addition, the dose-dependent elevation of free AA is also noteworthy (Figure 8). Diacylglycerols were relatively variable (13 identified molecular species), and those with carbon numbers of 32 and 34 (i.e., in their FA side chains) were depleted (Appendix Table_S03).

**Figure 8:**
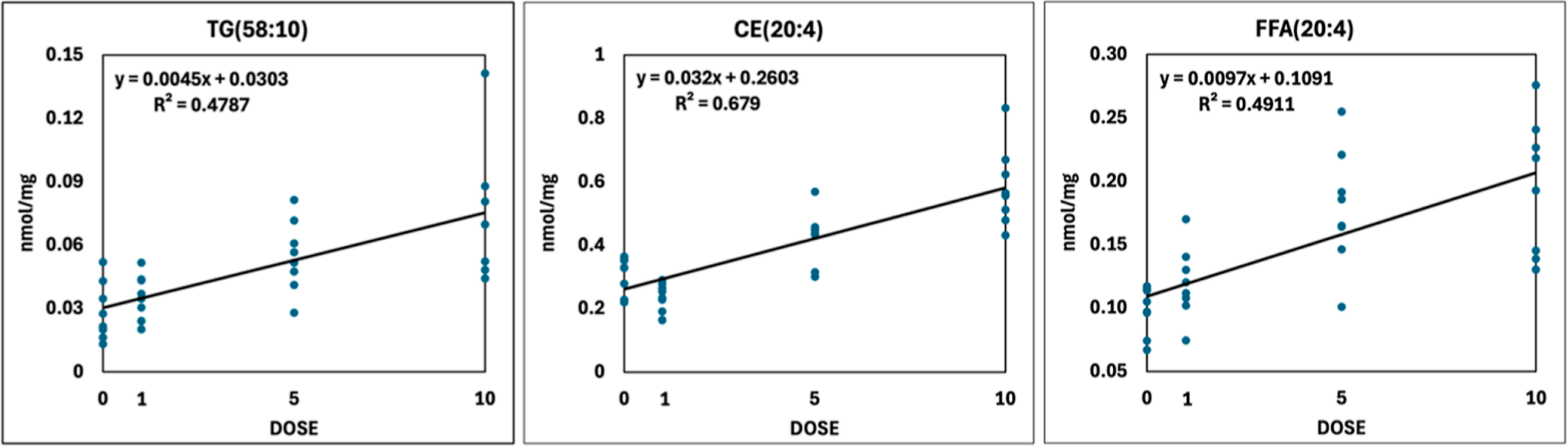
Linear dose–response of AA-containing neutral lipid molecular species in the triglyceride (TG), cholesteryl ester (CE), and free fatty acid (FFA) classes.

### 3.5 Relationship between Lipidomics Data and Histopathological Findings

For a more detailed understanding of histopathological alterations, the link among the identified histological changes and the lipidomic data set was tested. The Spearman rank correlation coefficients between the most abundant lipid molecular species in a given lipid class and the distinct histopathological changes are shown in Table 2. The total correlation data set for all lipid molecular species can be found in the Appendix Table_S10.

**Table 2:**
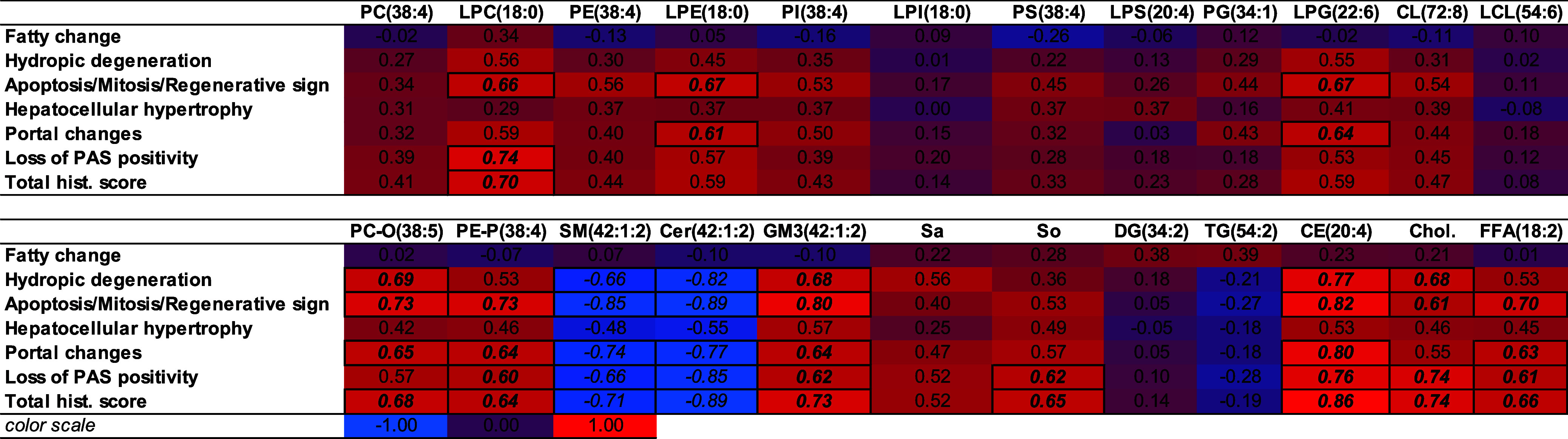
Spearman Rank Correlation Coefficients between the Identified Histological Changes and the Most Abundant Lipid Molecular Species of Each Lipid Class^a^

a Values below and over −0.6 and 0.6, respectively, are marked.

The steatotic fatty change did not provide a robust positive correlation with any of the lipid classes. Hydropic degeneration was positively associated with PC-O(38:5), GM3(42:1:2), CE(20:4), and free cholesterol, while it was strongly negatively related to Cer(42:1:2) and SM(42:1:2). We found markedly positive correlations between the severity and prevalence of apoptosis/mitosis/regenerative signs with the most abundant species of the LPC, LPE, LPG, PC-O, PE-P, GM3, CE, and FFA classes, and free cholesterol, while SM and Cer species (e.g. 42:1:2) were in a strong negative relationship with this change. Hepatocellular hypertrophy did not provide a strong correlation with any of the abundant lipids. Portal changes exhibited generally similar lipid associations like apoptosis, while the loss of PAS intensity (i.e., glycogen depletion) and the total, cumulative histological score were positively associated with LPC, PC-O, PE-P, GM3, CE, and FFA abundant species, and additionally with cholesterol and sphingosine, while negatively associated with SM (42:1:2) and Cer (42:1:2).

Furthermore, for the two toxicologically most relevant hepatic histopathological changes, the top 20 rank correlation coefficients are plotted in Figure 9, highlighting the plausible biomarker lipid molecular species. In both cases, ether lipids and shorter-chain sphingolipids displayed positive associations, while longer-chain Cer and SM species showed negative correlations.

**Figure 9:**
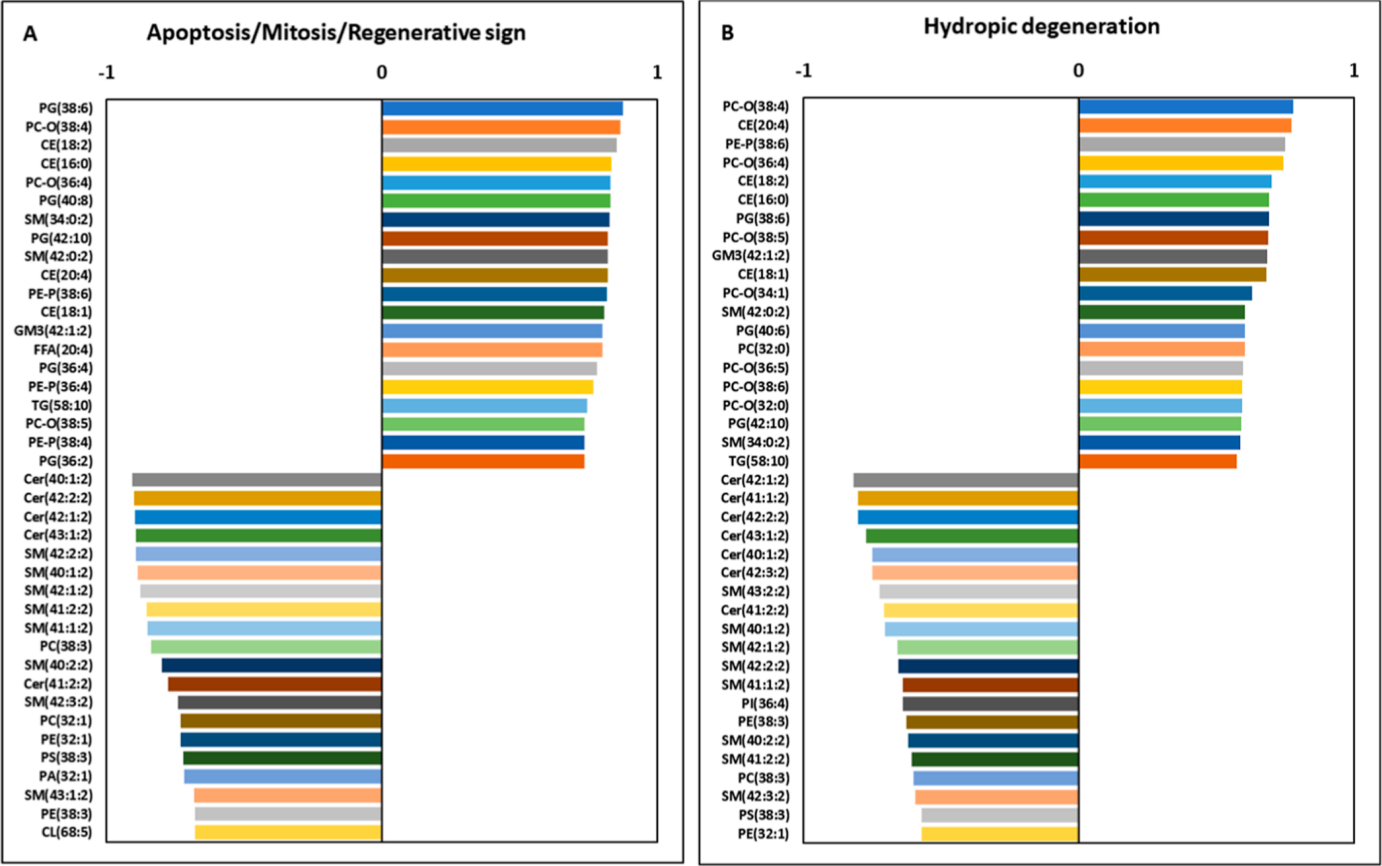
Top 20 strongest positive and negative Spearman correlation coefficients among (A) apoptosis/mitosis/regenerative sign and (B) hydropic degeneration and the individual lipid molecular species.

### 3.6 Correlation of Clinical Chemistry Results with Lipidomics

Clinical chemical endpoints were tested for Pearson correlation with the most abundant lipid species of each lipid class (Table 3). Individual molecular species correlations are shown in Appendix Table_S09.

**Table 3:**
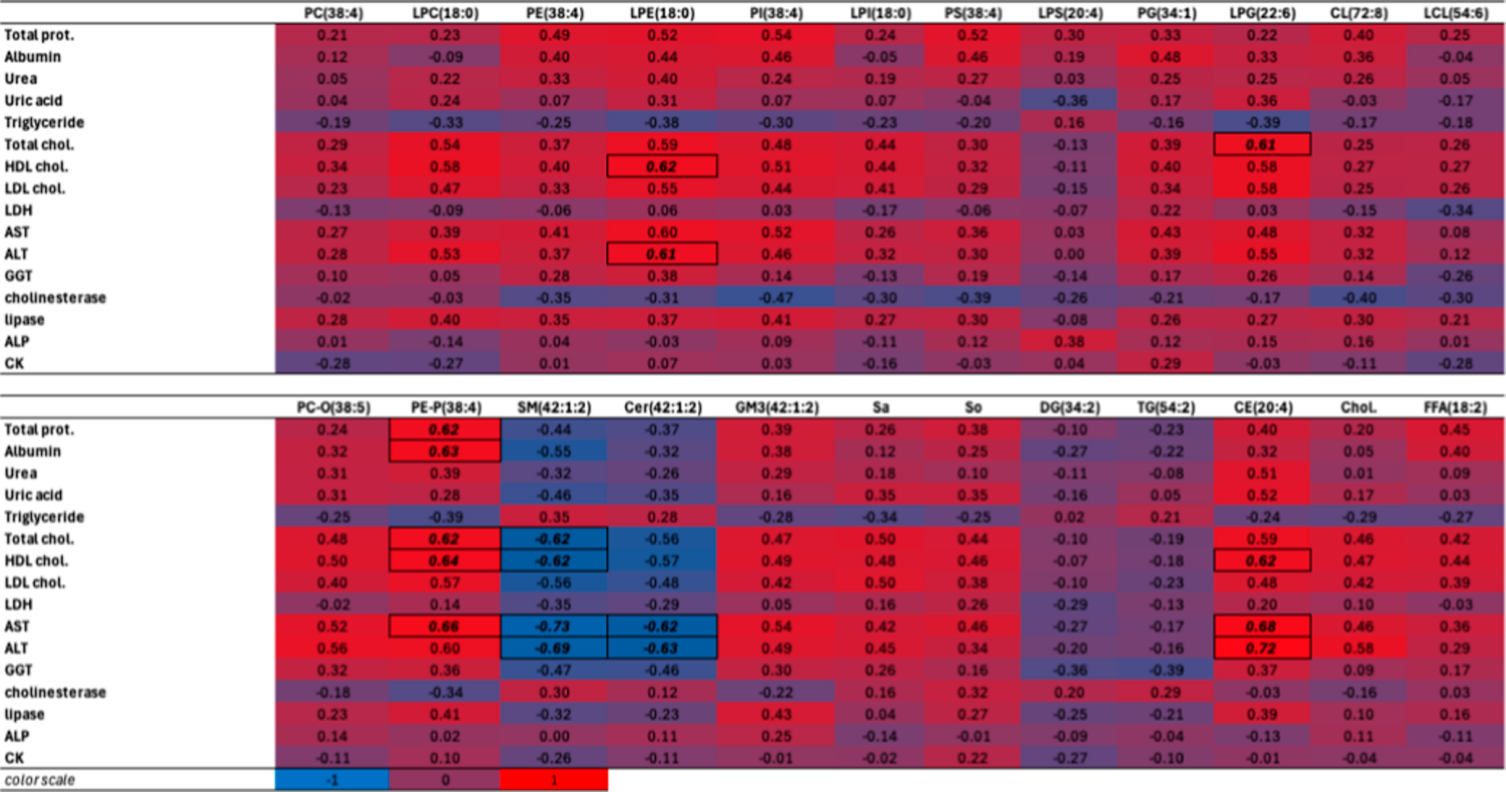
Pearson Correlations among the Plasma Clinical Chemical Parameters and the Most Abundant Hepatic Lipid Molecular Species of Each Lipid Class^a^

a Values below and over −0.6 and 0.6, respectively, are marked.

Setting the cutoff value to 0.6 and interpreting only hepatic and FB1 toxicologically relevant results, total and HDL cholesterol provided strong positive relationships with LPE(18:0) and LPG(22:6), respectively, and both were positively related to PE-P(38:4) as well; interestingly, SM(42:1:2) showed the opposite relation. Total cholesterol was positively correlated with CE(20:4), being a building block. AST, one of the most widely accepted biomarkers,^48^ was in a positive relationship with LPE, PE-P, and CE abundant species, while SM and Cer were in an opposite relation with this enzyme. For these two well-known biomarkers of FB1-induced hepatotoxicosis, the top 20 positively and negatively correlating lipid species were filtered (Figure 10A and B).

### 3.7 Transcriptomics (and Lipidomics Correlations)

To analyze FB1 dose-dependent gene expression alteration patterns, first, linear regression was performed against the FB1 dose, followed by enrichment analysis with the involvement of genes where the determination coefficient of the model exceeded 0.5. The results were ranked based on the slope of the regression models (beta) (Table 4).

**Table 4:** Top 20 Down- and Upregulated Genes in the Liver (Beta is the Slope of the Regression Model Set Against FB1 Dose, Fitting was Performed on 4 × 8 Individual Data)

rank order	DOWNREGULATED	beta	UPREGULATED	beta
1	LOC103693974	–0.43	**Mmp12**	0.65
2	Lgsn	–0.35	LOC102549755	0.53
3	Stac3	–0.31	Abcb1b	0.51
4	**Pla2g2a**	–0.31	**Trem2**	0.50
5	LOC102547849	–0.29	Cyp2c24	0.48
6	LOC102551428	–0.28	Atf3	0.46
7	LOC100910106	–0.28	LOC103693477	0.42
8	Inmt	–0.28	Mmp3	0.41
9	LOC102553657	–0.28	**Ccl2**	0.41
10	**Pnpla3**	–0.27	Tex36	0.40
11	Clic3	–0.27	Gpnmb	0.39
12	LOC102553540	–0.26	LOC102555660	0.39
13	LOC102551365	–0.26	Bcat1	0.39
14	LOC103690303	–0.25	Maff	0.37
15	Cyp26a1	–0.24	Spp1	0.37
16	LOC102547310	–0.24	Neurl3	0.36
17	Papss1	–0.23	**Cxcl10**	0.36
18	Prss55	–0.23	**Pla2g2d**	0.35
19	Per2	–0.22	Ccl1	0.35
20	Olr1305	–0.22	Sfn	0.34

Among the top 20 upregulated items, we identified phospholipase and triglyceride lipase encoding genes, but more importantly, signs of FB1-induced immunomodulation could be recognized, such as the upregulation of Mmp12 (macrophage metalloelastase), Trem2 (the triggering receptor in immune cells), and Ccl2 and Cxcl10 (chemokine ligands) (bold typing in Table 4). Checking the AA-associated pathways, prostaglandin D2 (Ptgds) and leukotriene 4 (Ltc4s) synthase, as well as arachidonate 12-lipoxygenase (Alox12), were downregulated, while the cytochrome P450 family member (Cyp2c55) was upregulated (involved in AA and LA epoxygenase activity regulation).

Figure 11 A and B plot the gene ratios of the down- and upregulated processes based on the Gene Ontology (GO) database. We found numerous upregulated genes related to apoptosis and inflammatory processes. Consistent with this, the cluster heatmap for the top 100 immune-related genes (Figure 12) indicates that FB1 induced changes at the level of transcription, but it was sizable only at and above the 5× dose.

**Figure 10:**
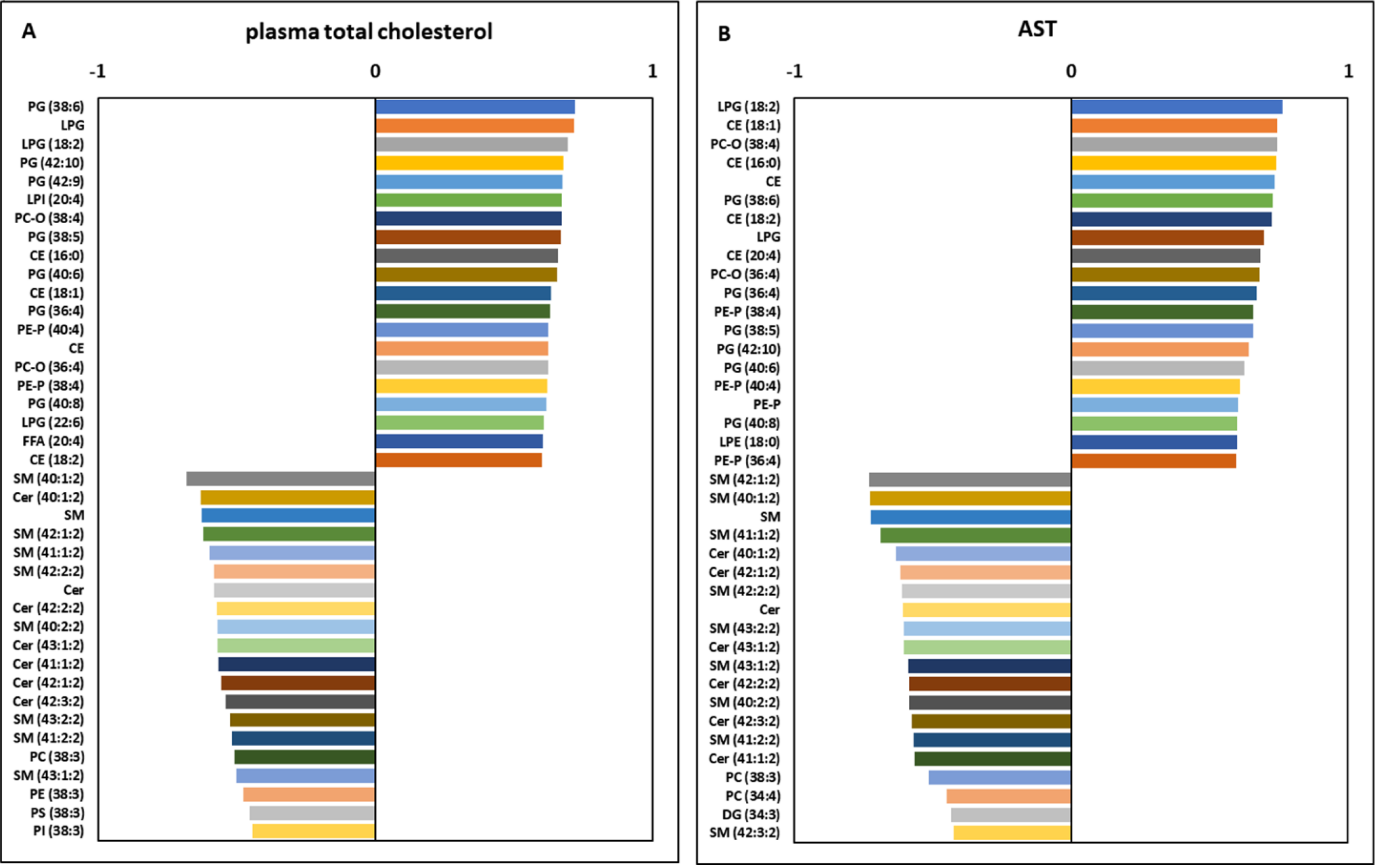
Top 20 positively and negatively correlating lipid molecular species’ Pearson coefficient values for (A) plasma total cholesterol concentration and (B) AST activity.

**Figure 11:**
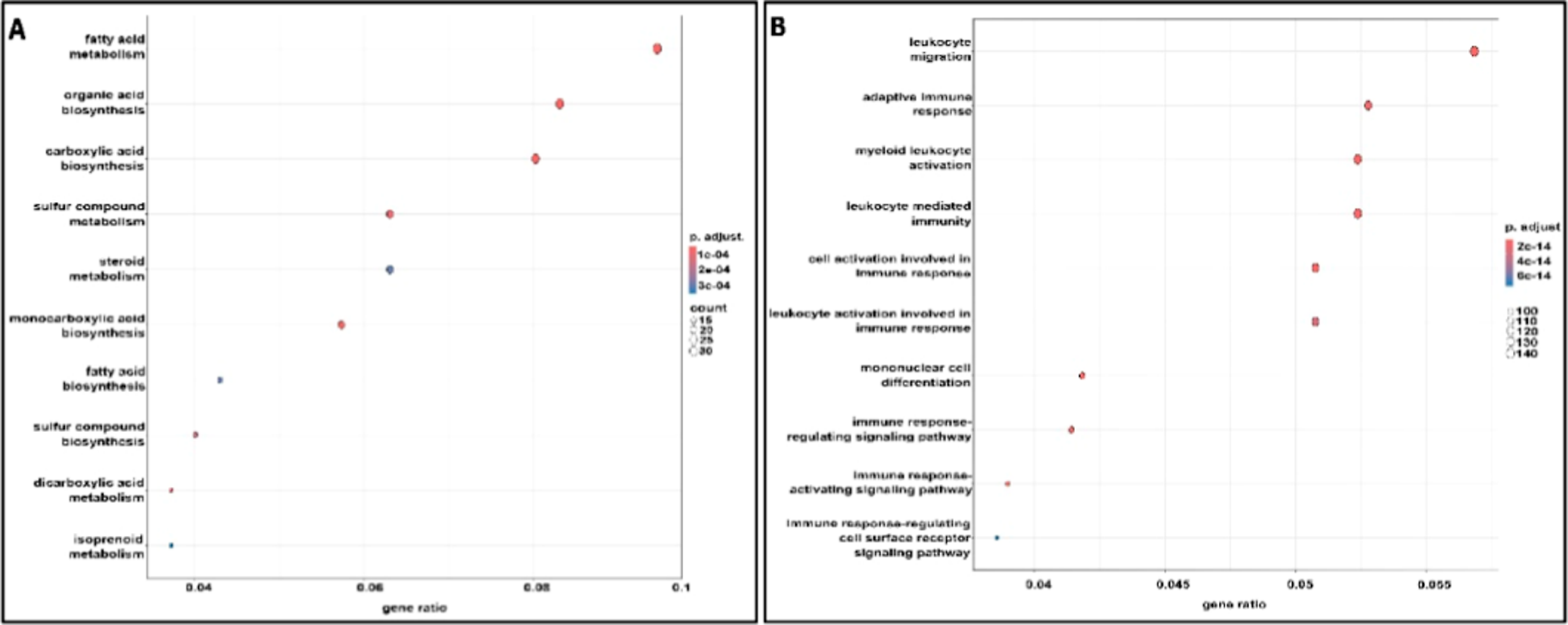
Top 10 most important biochemical processes downregulated (A) or upregulated (B) in rat livers by FB1.

**Figure 12:**
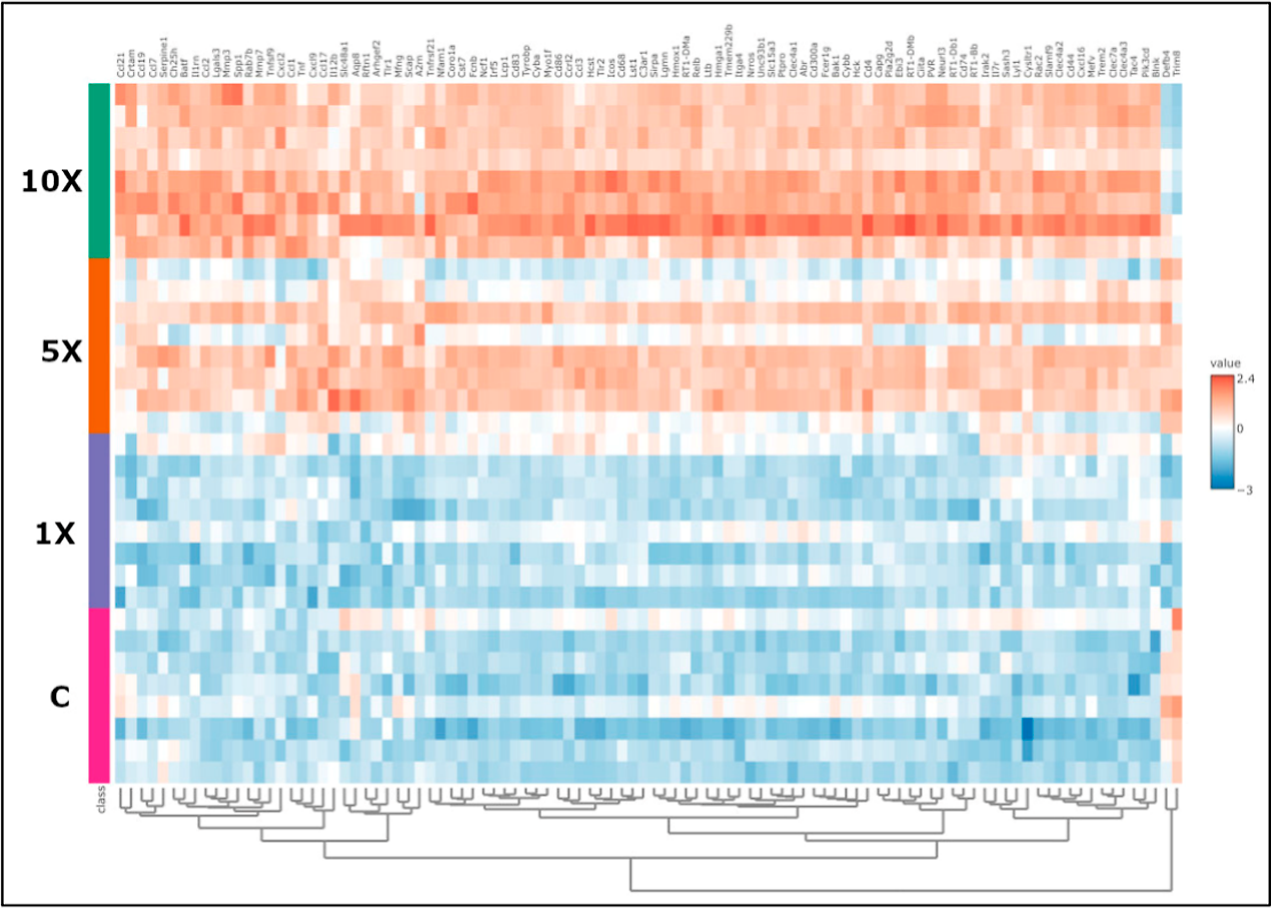
Hierarchical clustering based on the top 100 immunologically competent genes.

The top 20 correlation values between lipidomics and transcriptomics data provided evidence for the outstanding importance of Cer, SM, PG, and PC-O species (Table 5 and Appendix Table_S16).

**Table 5:** Top 20 Positive and Negative Correlations among Lipid Molecular Species and Individual Genes

rank order	lipid species	gene	corr. coeff.	lipid species	gene	corr. coeff.
1	**Cer**(42:3:2)	Mmp12	–0.9523	**PG**(38:6)	LOC102549755	0.9226
2	Cer(42:2:2)	Mmp12	–0.9422	**PG**(38:6)	LOC103693477	0.9200
3	SM(40:1:2)	Pla2g2d	–0.9281	**PG**(38:6)	Pla2g7	0.9043
4	**Cer**(42:3:2)	Slc2a6	–0.9252	PC-O(38:4)	LOC102549755	0.9041
5	**Cer**(42:3:2)	Irf5	–0.9237	PG(40:8)	C3ar1	0.8992
6	Cer(42:2:2)	Relb	–0.9226	**PG**(38:6)	Cyp2c24	0.8946
7	SM(42:1:2)	Pla2g2d	–0.9225	**PG**(38:6)	Gpnmb	0.8946
8	**Cer**(42:3:2)	Cd83	–0.9191	**PG**(38:6)	Abcb1b	0.8945
9	Cer(41:1:2)	Cd5l	–0.9189	**PG**(38:6)	Lgals3	0.8912
10	**Cer**(42:3:2)	Relb	–0.9175	**PG**(38:6)	Iglon5	0.8902
11	Cer(41:1:2)	Mcm2	–0.9162	PG(40:8)	Cyp2c24	0.8893
12	Cer(42:1:2)	Cd5l	–0.9152	**PG**(38:6)	Clec4a3	0.8879
13	sum_Cer	RT1-Bb	–0.9144	PC-O(38:4)	LOC103693477	0.8837
14	Cer(42:2:2)	Cd83	–0.9143	PC-O(38:4)	Gpnmb	0.8831
15	Cer(42:1:2)	Mmp12	–0.9138	PC-O(36:4)	Gpnmb	0.8827
16	SM(42:1:2)	Capg	–0.9122	**PG**(38:6)	Clec7a	0.8818
17	sum_Cer	Cd5l	–0.9120	PC-O(36:4)	LOC103693477	0.8806
18	**Cer**(42:3:2)	Bcat1	–0.9117	PC-O(38:4)	Neurl3	0.8792
19	**Cer**(42:3:2)	Cd5l	–0.9105	**PG**(38:6)	Ebi3	0.8779
20	SM(40:1:2)	Capg	–0.9102	**PG**(38:6)	Neurl3	0.8778

Major longer chain (40<) Cer species were negatively correlated to genes involved in immunomodulation (Mmp12, Irf5 (interferon regulatory factor), RT1-Bb, Capg, and Pla2g2d (secreted PLase having immunomodulatory effects)), or NF-kB pathway-related genes (RelB, CD83), glucose metabolism (Slc2a6), apoptosis regulation (Cd5l), or mitochondrial amino acid metabolism (Bcat1). From the top 20 list, 7 cases were associated with Cer(42:3:2).

Phosphatidylglycerol and ether PC-O molecular species were strongly positively correlated to genes regulating immune response-inflammation (Clec4a3, Neurl3) and tumor suppression (Arl11) or both processes (C3ar1), cellular immune response (Clec7a, Pla2g7 involved in LDL hydrolysis, Ebi3), or to genes having cancer association (Gpnmb) or involvement in xenobiotic metabolism (Cyp2c24). The most important lipid species of the highest representation was PG(38:6) (12 cases out of 20). For the over-represented lipid metabolite-correlated genes, GO analysis was performed for the description of their biochemical impact (Figure 13).

**Figure 13:**
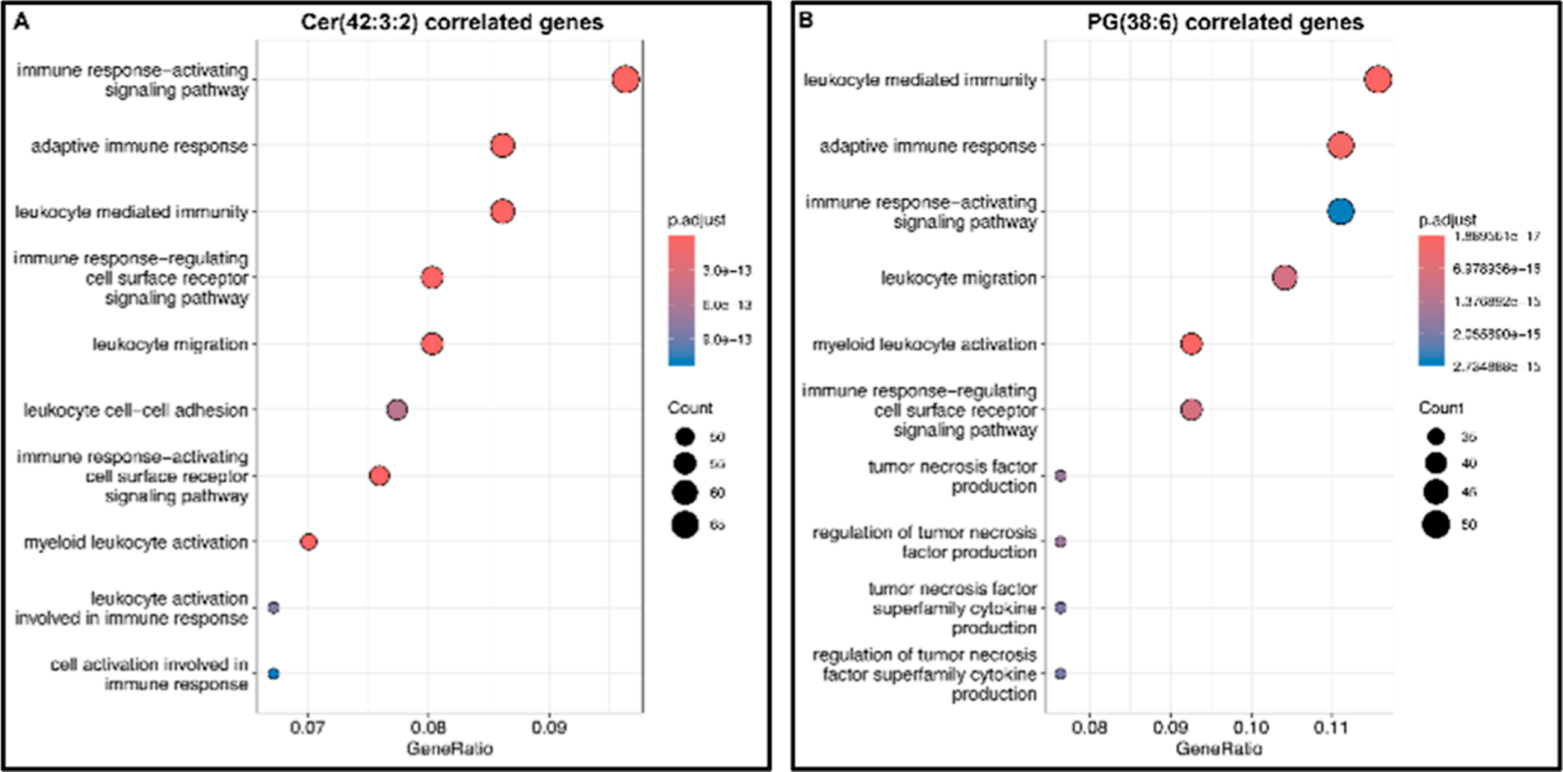
Top 10 biochemical processes regulated by genes in strong correlation with Cer(42:3:2) (A) and PG(38:6) (B), as assessed with GO analysis.

The ontology analysis of genes with a strong negative correlation with Cer(42:3:2) covered exclusively immunological processes related to adaptive immunity. The regulation of the immune response activation pathway, that of the adaptive immune response, and the leukocyte-related processes (migration, cell surface receptor signaling) involved at least 50 individual genes (Appendix Table_S17). Major processes regulated by genes in positive correlation with PG(38:6) were of similar roles, i.e., participating in adaptive immune response. Additionally, a smaller cohort of genes in this relatedness was involved in the TNF superfamily cytokine production and its regulation (gene count ∼ 20). PC-O(36:4) was also over-represented as a metabolite positively correlating with genes involved in the exact processes indicated for Cer(42:3:2).

The mostly affected processes and their regulation have been further analyzed by means of the Pathview application,^45^ mapping the differently expressed genes onto the respective biochemical pathways, as assessed from the KEGG database. We present data for comparison of the control and 10× groups. The GO enrichment analysis identified the downregulation of steroid biosynthesis, which is a novel finding. Figure 14 provides information about the gene expression changes in the steroid biosynthesis (*C* vs 10×), efficiently marking the downregulation of the genes Cel (carboxyl ester lipase) and Dhcr7 (7-dehydrocholesterol reductase), key components in cholesterol and CE synthesis.

**Figure 14:**
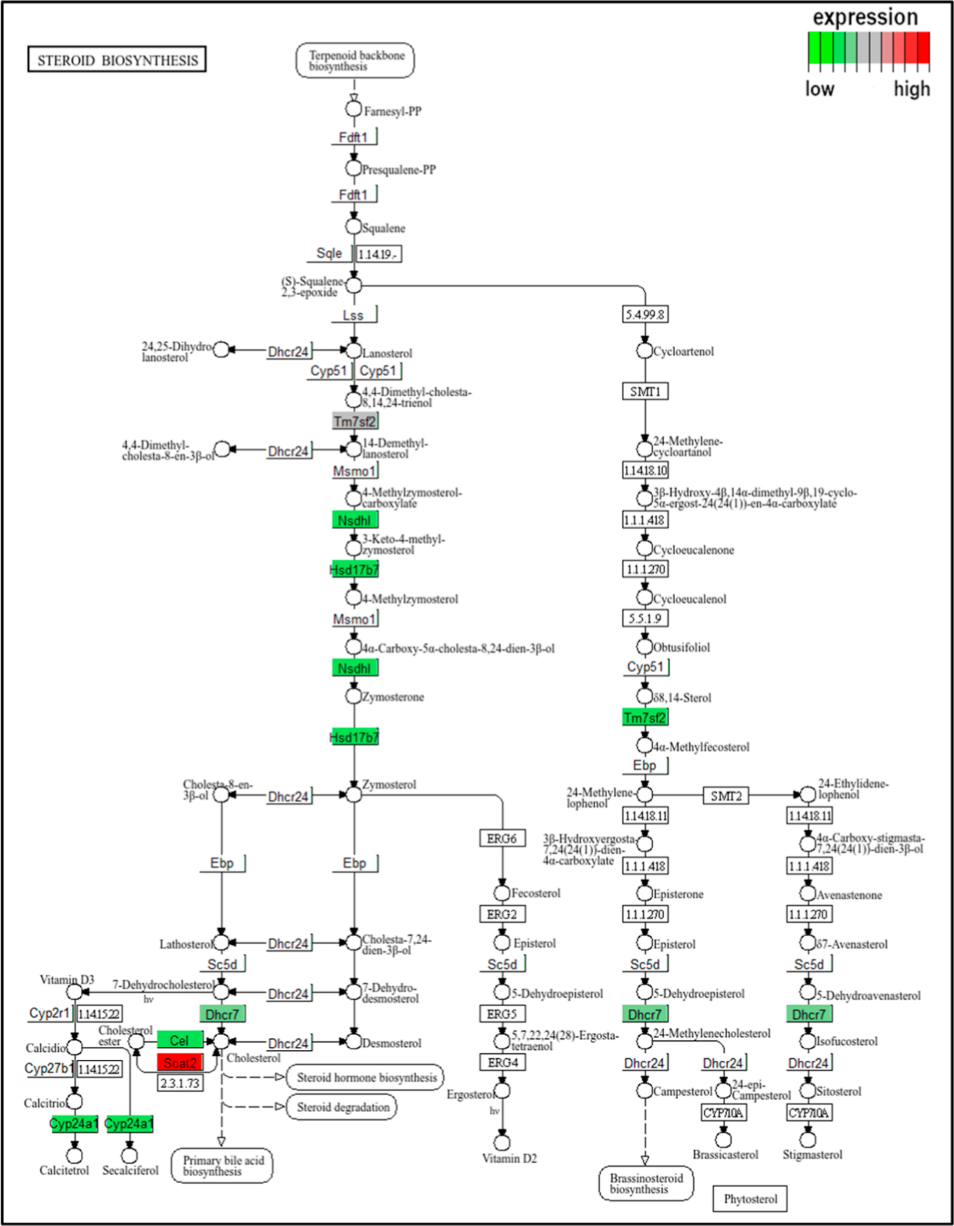
Modulation of the steroid biosynthesis (C vs 10× level FB1 treatment, mapped with Pathview on a KEGG pathway).

Enrichment analysis identified inflammatory and apoptotic processes among the over-represented GO terms in the 5× and 10× groups. In Figure 15, the KEGG pathway analysis (*C* vs 10×) highlights the upregulated canonical extrinsic and intrinsic apoptosis pathways. In parallel, we observed the activation of the TNF, MAPK, NF-kB, and PI3K-Akt signaling pathways and the granzyme B pathway, indicating the immunomodulatory effect of FB1. The image efficiently marks the pro-survival and pro-apoptotic genes (lower right segment) and suggests the nuclear membrane integrity loss via the upregulation of Gzmb. On the other hand, the image illustrates the absence of a DNA-damaging effect of FB1.

**Figure 15:**
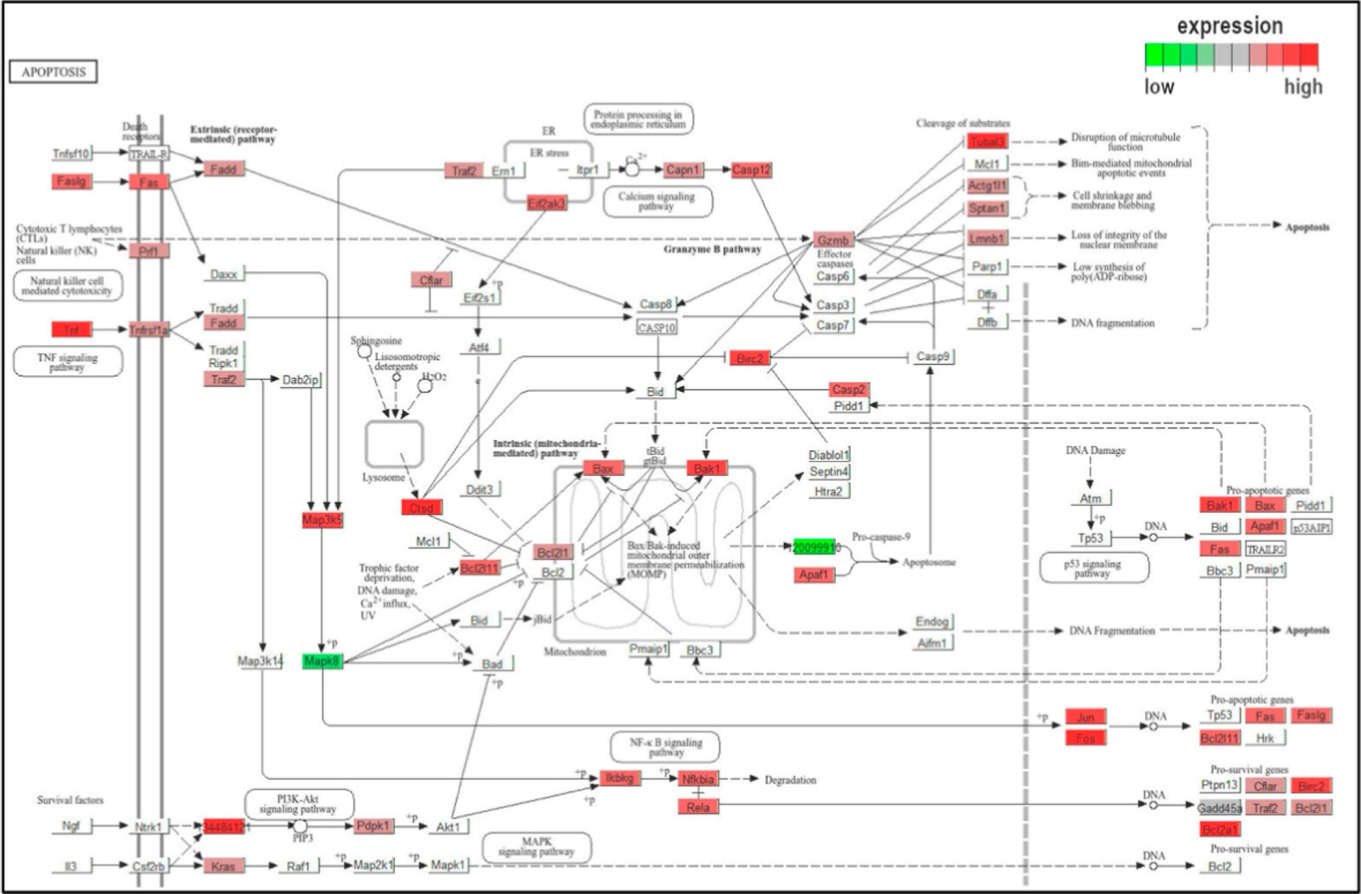
FB1-induced modification of the regulation of apoptosis in the rat liver. (C vs 10× FB1 treatment, mapped with Pathview on a KEGG pathway).

## 4 Discussion

### 4.1 Somatic Traits (FB1 Dose and Exposure Time Dependence)

As a cumulative outcome of the reduced feed intake and organ weight decrease, BW gain provided a dose-dependent decrease for FB1 (Appendix Table_S02). Retarded growth was also observed in rats in previous FB1 studies of short exposure (less than the clearance time for rats^19^) but higher doses (0.75 mg/kg BW, i.p. for 2, 4, or 6 days;^49^ 20–50–100 mg/kg diet for 5 and 10 days,^14^ sometimes exceeding the hepatic clearance time^19^); we implement our results assuming possible (yet undetermined) hepatic FB1 accumulation. The most recent findings to explain lower BW, decreasing BW gain, and organ weight revealed decreased metabolic activity of jejunal myenteric neurons and a markedly (by 38%) decreased hepatic mitochondrial metabolism, exerting a negative impact of FB1 on the neuroplasticity of the enteric nervous system in rats.^50^

### 4.2 Clinical Chemistry

Within nitrogenous compounds, the increase of total protein and albumin at the 10× (∼16 mg/kg dietary dose eq.) is in accordance with our earlier rat results^14^ at 50 mg/kg dose and with those at 250 mg/kg.^51^ We assume partly the immune system involvement/activation here (globulin fraction within the total protein), as also revealed by transcriptomics (Figure 11B), since the calculated globulin concentration also exhibited an increase, extending the total protein concentration increase to the extrahepatic site, the B cell-mediated globulin synthesis. Within the serum lipids we propose two key assumptions regarding serum cholesterol increase: the downregulated steroid synthesis (transcriptomics data) and the markedly decreased liver weight. These conditions together suggest an FB1-induced alteration in cholesterol redistribution. One of the most compelling confirmations of this mechanism was provided by Merrill et al. (1995),^52^ who reported that FB1-treated rat hepatocytes in culture secreted apoB, PC, and cholesterol, even when their sphingolipid synthesis was blocked, thereby resulting in the absence of Cer and SM in the secreted VLDLs.

### 4.3 Histopathology

The histological investigations identified typical hepatotoxicosis at the 5× and 10× levels. Signs of apoptosis are consistent with the findings at 35–75 mg/kg dietary dose.^53^ According to our data, these changes were located around the central vein, as it was also observed at 10 mg/kg; the authors also reported rapid apoptosis development as early as 24 h post injection (p.i.).^54^ Mitosis, the typical compensatory response to replace apoptotic cells, occurred simultaneously with apoptosis, agreeing with results in F344 rats at 250 mg/kg FB1 for 21 days.^51^ Another characteristic symptom was hydropic degeneration, also termed ballooning. Deregulation of the hepatocyte osmotic balance leads to swelling and vacuolization of the mitochondria, cytosol swelling, and water entering the cells,^55^ which is associated with the activity change of ion pumps.^56^ In contrast to Moon et al. (2000),^54^ we did not detect vacuolation, while the full absence of necrosis agreed with their findings; the authors furthermore observed hydropic degeneration as early as 3 h p.i., and we showed that it persists for up to 5 days, but only above dose 1×. Moreover, the mild but apparent lymphocytic infiltration and inflammatory process are also well supported by earlier results, particularly regarding the presence of sinusoidal macrophages.^54^ The observed loss of PAS staining positivity, typically localized pericentrally, indicates cellular glycogen content depletion, in agreement with our recent findings, where FB1 increased the hepatic carbohydrate uptake rate.^57^ We note that FB1-induced hepatic glycogen depletion had not been systematically demonstrated before, only hypothesized (in pig^35^ and rabbit liver^58^). In a recent study, human hepatocytes provided an analogous reaction upon xenobiotic treatment, which could be efficiently reversed by a glucosyl-ceramide synthase inhibitor, leading to the reduction of cellular GM3 ganglioside levels.^59^

### 4.4 Liver Lipidomics

#### 4.4.1 Sphingolipids and Ceramide Synthases

From a sphingolipidomic viewpoint, the mechanism of action of FB1 is well-known and is based on the inhibition of Cer synthases, subsequent Cer synthesis inhibition and marked precursor (Sa, So) accretion.^60,61^ At the gene expression level, the characteristic FB1-induced inhibition of Cer synthesis could only be proven at the 10× dose for CerS2–4, which transfers very long-chain FAs (C22–C24) onto the C18-long sphingoid base, thereby producing longer-chain (*C* > 40) Cer species; this aligns with a biomarker indication,^62^ pointing to C22–C24:C16 sphingolipids’ ratio. Our lipidomic data set partly confirms these results, as the concentration of such Cer species (e.g., the most abundant Cer(42:1:2)) decreased in the 10× group, and we also detected a significant reduction in the corresponding upstream SM species (SM(42:1:2)). Tardieu et al. (2021) published relevant results on the hepatic sensitivity of chickens, where 20 mg/kg FB_1+2_ (“a dose considered as safe”) efficiently perturbed the sphingolipid profile.^63^ Though CerS expression was not analyzed by Lassallette et al. (2023), the authors reported increasing tendencies in the dihydro-Cer molecules 18:0/22:0, 18:0/23:0, and 18:0/24:0 in FB1-treated chicken livers.^62^ In that avian study, hepatic C24 to C26 ceramides provided unchanged or increased concentrations, showing only partial agreement with our data, where the only Cer-42 molecular species unchanged was Cer(42:0:2), while the unsaturated forms were depleted. In rodents, total SM concentration decrease was also reported upon FB1 exposures at comparable or higher than the 10× dose both in vivo^64^ and in vitro (primary hepatocyte lipid rafts;^65^ Chang cells;^66^ primary hepatocytes and HepG2 cells^67^). On the other hand, sphingolipid synthesis inhibition by FB1 seems to depend only partially on genetic regulation (and may manifest mostly on an enzymatic level). The significant increase in several Cer species (Figure 6A), including the most abundant component, at the 1× level does not align with the unchanged gene expression pattern. More specifically, the expression of CerS1, which synthesizes shorter-chain Cers, elevated at higher FB1 concentrations, we suppose species-specific characteristics and distribution of CerS isoforms behind the results.^68^ Although Cer(34:1:2) was not changed, these data might support the elevation of the upstream SM(34:1:2). Importantly, in most cases, the observed changes of SL species, either decreasing or increasing, provided strong linear FB1 dose dependence.

In the frame of data evaluation, we were seeking proof of possible adaptation against FB1-induced apoptosis induction, since the development of an “anti FB1 lipid profile” was indicated in rat liver; this is characterized by lower C18:0 and higher total monounsaturation in the SMs as an antiapoptotic defense reaction.^69^ We do not suppose anti-FB1 defense development in the present study in the SMs after 5 days, as the shorter SM species (C34–36, most probably integrating stearic and palmitic acids) typically increased, and the monounsaturation (in longer chain SM species over C36) decreased systematically.

#### 4.4.2 FB1 Impacts Glycerophospholipid Metabolism

In addition to the affection of sphingolipids, the first results reporting the FB1-induced rearrangement of the glycerophospholipidome were likely published in Fisher rats at 250 mg/kg dietary dose for 21 days.^67^ Authors observed an increase in the PE/PC ratio in subcellular membrane lipids’ composition. Later similar findings were reported in the lipid rafts of primary rat hepatocytes in vitro.^65^ The PE/PC increase refers to elevated membrane rigidity, a change considered to promote cancer development. In our study, we also detected a significant increase in the PE/PC, or rather in the PE/(PC + PI) ratio, due to the increase of PE already at the 1× dose.

In addition to the major lipid classes, we detected numerous alterations in the minor lipids. PG and BMP (bis­(monoacylglycerol)­phosphate) account for 1–2% of phospholipids in most animal tissues and display versatile but fairly different functions in cells, being mitochondrial and lysosomal signature lipids, respectively. A recent work has mapped their tissue distribution, proving the presence of both structures in rodent liver.^71^ Because the liver is a lysosome-rich organ, where BMP lipids are particularly abundant and are represented by highly unsaturated species, the dose-dependent accumulation of likely BMP species, such as BMP(42:10) or BMP(44:12), might represent the onset of autophagy due to FB1. PG is the biosynthetic precursor of CL, the hallmark lipid of the mitochondrion. Therefore, the increases in the less unsaturated and likely PG species (34:1 or 36:4) seem to imply deteriorated CL synthesis and suboptimal mitochondrial function. Interestingly, we observed an opposite trend, i.e., the level of CL elevated following a logarithmic pattern. Additionally, the CL profile, which is dominated by the symmetric, tetra-linoleyl species (CL­(72:8, tetra18:2)) in the rat liver (60% of the CL content), moved toward an even more symmetric pattern (Figure 7), favoring proper mitochondrial function; thereby, it might represent a counteracting/adaptation effect to FB1 treatment.

PE-Ps (PE plasmalogen, PE with a vinyl ether bond in the *sn*1 position) are considered to have antioxidant and antiapoptotic properties,^72^ whereas plasmanyl PCs (PC with an ether bond in the *sn*1 position) are known to be sensitive markers of tissue injury (acute kidney injury^73^), xenobiotic effects (e.g., in fish muscle lipidomics^74^), or cancer aggressiveness.^75^ Resolving the FA chains of the PE-P species in our study, those with marked concentration change were of C18:2n6 and C20:4n6 types, while n3 acyl chains (C20:5n3 and C22:5n3) were less increased; the former are eicosanoid precursors, and the latter are resolvin and protectin precursors.

PC-Os’ (ether type PC-s) systematic accretion refers to increased peroxisomal activity,^73^ but in our direct histological evidence could not be established. These lipids act in apoptosis control as well, maintaining cell viability.^73^ In PC-Os we found increases in all molecular species at dose 5× and above, referring to a non-FA-specific accretion; this might be a protective/compensatory (possibly antiapoptotic) effect.

One of the most prevalent and novel findings in the liver was the GPLipidome-wise accumulation of AA-containing lipid molecular species. The most affected species were the major structural components PE(38:4) and PI(38:4), which provided logarithmic-type increases with significant changes already at dose 1×, while the elevations in the ether lipids PC-O(38:4) and PE-P(38:4), in the neutral lipid CE(20:4), as well as free AA, followed linear dose dependence. Consistent with this, AA accumulation was detected in the total phospholipid fraction in piglets’ livers at a 20 mg/kg FB1 dose after 10 days.^35^ It was also shown that a high-fat diet promoted the increase in AA levels, especially in the phospholipid fraction, along with upregulated inflammatory processes.^76^ Furthermore, several AA-containing species, such as PC-O(38:4), PE-P(38:4), PG(40:8), or CE(20:4), provided strong positive association with histological changes (apoptosis/mitosis and glycogen depletion) as well as with plasma cholesterol and hepatocellular enzyme levels, indicating the importance of AA in these changes. Moreover, these AA-containing ether lipid species were strongly correlated to the lipid species showing top association to the transcriptomic data set (Table 5 and Appendix Table_S16), highlighting their possible association with the inflammatory regulation. Murphy and Folco (2019)^77^ reviewed the pathways where AA is hydrolyzed from the membrane lipids (by PLA2) and where it enters leukotriene synthesis (LTs A4, B4, and C4). Authors focused on the role of the Lands cycle as a means to incorporate “extra” AA into membrane PLs. In this process, AA is a substrate of lysophospholipid acyltransferases (LPCATs), being in part AA-specific (LPCAT3), but rather a long-chain-fatty acyl CoA synthase (ACSL) is the enzyme providing exclusive preference toward AA. Kuwata and Hara (2019)^78^ reviewed the possible role of ACSL4, an AA-specific acyl-CoA synthetase enzyme, and cited a study where this was upregulated in human hepatocarcinoma cases.^79^ The upregulation has been explained with a mechanism to deplete unesterified AA (and to esterify AA into GPLs), being a direct antiapoptotic action. We keep the assumption that the channeling of AA was not exclusive toward the synthesis of eicosanoids (meanwhile Tbxas1, Cyp2c55, and Ptgs were upregulated), as CE(20:4) also increased in parallel with the FB1 dose (referring to increased Acyl-CoA cholesterol acyl transferase activity, ACAT1). Hepatic enzymes and regulatory metabolites involved were highlighted in a short (4–9 days) FB1 study on chickens.^36^ A marked increase in epoxide-type, P450 enzyme-derived oxylipins was reported after 4 days and, interestingly, in a less FA-specific and FA-type (n3 or n6) manner. The majority of oxylipins was C18 FA-derived, and the diol/epoxide ratios (pro-/anti-inflammatory indicator) were reduced, making inflammatory processes questionable. We conclude that the gradual AA concentration increase in the major GPLs may be a prerequisite of eicosanoid synthesis but may not strictly be the biochemical basis of inflammation (or mostly via the P450 route), as supported by Guerre et al., 2024.^36^ Highlighting the possible AA-based synthetic processes, we point out the intensification of diol-type oxylipin regulation and, in parallel, the down-regulation of the leukotriene synthesis. We think that the utilization of free AA in eicosanoid synthesis, in CE esterification, and most markedly via LCATs was speeded up by FB1, the latter probably as an antiapoptotic action.

#### 4.4.3 Neutral Lipid Lipidomics

Regarding neutral lipids, both free and esterified cholesterol concentrations increased in the 10× group concomitantly with the markedly increasing plasma total cholesterol levels and HDL and LDL cholesterol levels. FB1 treatment also increased the cholesterol content in rat hepatocellular lipid rafts,^65^ which has also been published for the rat hepatic microsomal and nuclear membranes,^81^ and for the hepatocellular plasma membrane.^69^ Possible explanations for these findings are complex, as we found organ mass decrease and cellular damage (histopathology and plasma enzymes) as well. FB1 exposure of HepG2 cells decreased the expression of the LDL receptor (Ldlr) while increasing the expression of the ATP-binding cassette transporter A1, leading to lower cellular lipid influx and higher cholesterol efflux.^83^ However, plasma total, HDL, and LDL concentrations increased in FB1-fed pigs after 14 and 21 days with unchanged Ldlr gene expression,^84^ which refers to unchanged hepatocellular LDL reuptake. Our gene expression data also revealed unchanged lipoprotein reuptake and downregulated steroid biosynthesis; therefore, we suppose cholesterol removal from one or more extrahepatic sites. We also note that the FB1-induced downregulation of steroid biosynthesis is a novel finding, involving the suppression of key genes of the cytochrome P450 superfamily.

### 4.5 Correlation of Clinical Chemistry Results with Lipidomics

Analyzing the possible relationship between clinical chemistry endpoints of high hepatotoxicological relevance and lipidomic data was reasoned to highlight possible lipid biomarker candidates. The most important association of circulating lipoproteins was found with the hepatic concentration of their most abundant building block, CE(20:4), but the role of the abundant molecular species of the LPE and PE-P classes has also been highlighted by correlation analysis (Appendix Table_09). Interestingly, the majority of PG molecular species provided a medium positive correlation with the circulating LP concentrations. The role of PGs is to act as minor and polar surface lipids of circulating LPs,^85^ but toxicological interference is questionable in our opinion. From the relevant and responsive enzymes in this study, we found AST to be positively related to PE-P, LPE, and CE molecular species. In these lipids and in those providing further strong positive associations (Figure 10B), by some molecular species the role of AA and linoleic acid seemed to be outstanding (e.g., PC-O(36:4) and LPG(18:2)), as also pointed out by Burger et al. (2018).^65^ Additionally, we highlight the importance of PE-P fractions here, with the molecular species containing linoleic acid and AA (PE-P(36:4), PE-P(38:4), and PE-P(40:4)). This predominant role of the n6 FAs, mostly AA, may be a segment of a more general (maybe regulatory) process, since CE and FFA fractions provided as well identical affection. In primary hepatocytes, nonesterified AA levels provide proliferation inhibitory and apoptosis induction effects.^65^ Additional precaution is needed when analyzing AST data in a lipidomic correlation context, since the KEGG pathway enrichment analysis (Appendix Table_S14) revealed down-regulation of the alanine, aspartate, and glutamate metabolism.

### 4.6 Relationship between Lipidomics Data and Histopathological Findings

The establishment of correlative links among histopathological changes and lipidomic data may reveal the biochemical background for the membrane destructive (total score correlations with lipidomic/membrane lipid data), apoptotic (most probably sphingolipid-mediated apoptosis), and immunotoxic (macrophage appearance) effects of FB1. Thus, in the further step, we were seeking lipidomics data associations. The most prevalent histologic finding was hydropic degeneration, providing a positive relationship with plasmanyl-PCs, GM3, CEs, and free cholesterol, while it was negatively related to Cer and SM species. The molecular species providing the closest correlation were mostly of n6 FA types with high abundance, e.g., PC-O(38:4) and PC-O(36:4), and CE(20:4), referring to the predominant importance of AA in these changes. Apoptosis occurred in parallel with hepatocellular proliferation; the strongest negative association of these changes was found specifically with longer chain SM and Cer molecules (40 and above), which is supported in NAFLD.^86^ We found contradicting correlations among Cer and SM molecular species and apoptosis; namely, unsaturation was not, but the number of hydroxyl groups was a differentiation factor. Those species without a hydroxyl group were positively correlated, while those having 1–2 or 3 were systematically negatively correlated to the histological change (Appendix Table_10). Guerre et al. (2022) published partly similar results to ours in fumonisin-fed turkeys’ livers, where C14–C16 ceramides decreased and C18–C26 species increased.^21^ These chain-length-specific changes were attributed to the CerS isoforms’ selective inhibition by FB1, according to the authors. An additional connection between apoptosis and SM molecular species has been reported,^88^ where SM(42:2:2) was found to significantly interact with hepatic insulin sensitivity during glycemic deterioration. We thus conclude that Cer and SM molecular species may induce apoptosis partly via the alteration of insulin signaling.^57,89^ The positive correlation of GM3 gangliosides with apoptosis has been earlier supported, reporting the role of GM3 in the postoperative regeneration of the liver.^91^ Since the GM3 ganglioside precursor is Cer, their opposite concentration change is logical. Authors as well reported a strong positive association of cellular GM3 levels with preoperative oxygenation, referring to the possible role of oxidative stress in this process. While we did not analyze oxidative stress markers here, oxidative stress was reported as the leading FB1-induced process in murine liver. This study highlighted the role of NF-kB showing agreement with our results, where the NF-kB signaling was among the top 5 up-regulated pathways in the liver.^92^

Of specific note may be the PAS staining intensity change, which was typically localized pericentrally. This finding indicates cellular glycogen content depletion. In a recent study, human hepatocytes provided an analogous reaction upon xenobiotic treatment, which could be efficiently reversed by a glucosyl-ceramide synthase inhibitor, leading to the reduction of cellular GM3 levels.^59^ This refers to the role of GM3 gangliosides (along with Cer) in the regulation of hepatocellular glucose uptake and cellular glycogen levels.

### 4.7 Transcriptomics (and Lipidomics Correlations)

The most affected pathways in FB1-induced hepatotoxicosis have been highlighted in pigs and mice,^84,93^ showing partial agreement with our findings (affection of the PI3K-Akt pathway and fatty acid metabolism). We report here a new and additionally strong FB1-induced disturbance of steroid biosynthesis (Figure 14, Appendix Table_S15), where the key genes (Cyp11a1 and Cyp11b1) were markedly downregulated, referring to the involvement of the cytochrome P450 superfamily enzymes. Disturbance at the biochemical level was manifested in the increased plasma concentration of lipoproteins, referring to an augmented release of those toward the extrahepatic sites. In agreement with Régnier et al. (2017),^84^ this was coupled with markedly downregulated FA biosynthesis (Figure 11A). To our current knowledge, this is the first study that gives a direct reference to the FB1-induced modification of FA biosynthesis, though it was proposed rather early.^94^ Additionally, FB1 exposure of pigs activated the Akt-PTEN pathway, thus slowing down hepatic lipid metabolism.^84^ Our results agree with this; within the PI3K-PTEN-Akt pathway, the expression of 66 genes was affected by FB1. The primary function of PTEN is its inhibitory action of the PI3K pathway, the latter regulating cell metabolism, survival, proliferation, apoptosis, and growth,^95^ thus PTEN having antitumor functions. Outstanding importance was indicated to the upregulation of this pathway in porcine liver^84^ since FB1 is known to alter the sphingolipid/ceramide signaling pathway as well (as proven here with 40 genes involved) that directly modulates PI3K-AKT signaling.

The GO analysis indicated hepatic immune response activation, primarily involving TNF and NF-kB pathways. Processes upregulated involved genes playing pivotal roles in leukocyte migration, adaptive and native immune function, cell activation, chemoattraction, and differentiation (e.g., Trem2 and Cxcl10). The simultaneous activation of both apoptotic and inflammatory pathways is consistent with our histological results and, importantly, aligns with the message of a recent review reporting that apoptosis and the apoptotic machinery can be rewired into an inflammatory process.^96^ Such changes at the gene expression level did not appear at the 1× dose, where lipidomics already revealed significant AA elevations, suggesting an additional and preceding regulation of metabolite levels, possibly at the level of enzyme activity (and not yet at the expression level).^84^ We suppose this especially for the AA-containing diacyl species (PE(38:4) and PI(38:4)) with log-type dose dependence and strongly increasing concentrations; their correlation with transcriptomics data mostly with linear dose–response is weaker and may refer to the early phase response of these lipid species manifesting before the transcriptomics reactions, directly at the enzymatic (proteomic) level.^84^ This study additionally highlights lipidomic associations with immunomodulation, where C40–41–42 Cer species (and in particular Cer(43:2:3)) provide determinant negative correlations with immunological processes, i.e., with their regulating genes; we interpret these associations (manifested at histologic and lipidomic levels) as a part of a hepatoprotective effect (arising from the inhibition and fallback of Cer and upstream molecules’ synthesis) against FB1. The potent anti-inflammatory effect against FB1 and the mode of action included modifications (fallback) of the neutrophil cell infiltration via decreased adhesion molecule-1 expression, increased TNF-a production, and propagation of apoptosis in the rat ileum;^97^ on a transcriptomic level, we found analogous hepatic results. The question why a relatively minor Cer species provided the closest correlation with the characteristic FB1-induced immune response genes may be intriguing, and we add that further Cer species of higher hepatic concentration were also indicative (e.g., the major Cer(42:1:2) and the second most abundant Cer(42:2:2)). However, we could not directly link the elevated AA level of the major GPLs to inflammation; this is partly supported by recent findings reporting pyroptosis and protective autophagy in FB1-induced enterotoxicity^98^ (as reported after 4 days in mice^99^ ), the latter supported by our positive PG (38:6) (BMP) correlations with transcriptomics results and also by the inflammasome-associated Nlrp1 upregulation.

In addition, we provide evidence for the upregulation of the carbohydrate metabolism (KEGG enrichment analysis), which also has a direct histopathological manifestation (glycogen depletion). This aligns with earlier results,^100^ describing the inhibition of hepatocyte gluconeogenesis by FB1. We propose a new link between the Cer molecular species and hepatic glucose metabolism, as supported as well by transcriptomics in FB1 treatment, which has not yet been highlighted in the most relevant study.^84^

## 5 Conclusions

Low-dose, short-term intraperitoneal exposure to FB1 and the combination of classical and omics-type methods represent an innovative approach to the investigation of hepatotoxicity in mycotoxin research. FB1 treatment evoked marked lipidomic profile alterations in the liver of male rats. This study newly revealed the perturbation of several GPL fractions (PE, LPE, PG, LPG, CL, PC-O, and PE-P), probably in relation with the apoptotic/mitotic processes and the hydropic degeneration of the hepatocytes but plausibly affecting lysosomal and mitochondrial lipids as well. Reactive lipid fractions may refer to the outstanding importance of AA in cellular reactions but did not undoubtedly confirm its role in inflammation; further research is needed to clarify the exact channeling of AA found in the major GPLs. The most important pathways affected were the PI3K-Akt-PTEN, the NF-kB, and the sphingolipid/ceramide signaling pathway (and proteomics would largely bridge the gap between transcriptome and lipidome). The establishment of the links between histopathological changes and lipidomic data reveals evidence for the membrane-destructive, apoptotic (likely sphingolipid-mediated), and immunotoxic effects of FB1. This study newly identifies downregulation of carbohydrate and steroid metabolism. From a clinico-chemical and histopathological viewpoint, the 1× dose is safe; however, lipidomics sensitively detected early biochemical reactions. Possible analytical limitations may arise from the characteristics of the shotgun approach (primarily due to ion suppression effects and coelution of isomers, which might prevent the detection of very low-abundance species and resolution of isomeric structures, respectively).

## Supplementary Material





## Data Availability

The data sets generated during the current study are available partly in the appendix file, while gene expression data in the GEO repository https://www.ncbi.nlm.nih.gov/geo/query/acc.cgi?acc=GSE286344, on request.

## Notes

(PDF)
Somatic data (bodyweight, feed intake, and organ weight), dose dependence regression model parameters, full lipidomics data set (nmol/mg wet weight), intergroup comparisons with *t*-test, linear and log dose dependence regression model parameters, fold changes of the hepatic lipid classes’ totals (nmol) in the 1×, 5×, and 10× groups, full correlation matrix of all lipidomics data, fatty acid composition of the rat diet, full clinical chemistry data set and intergroup comparisons with ANOVA, full data set of the hepatic histological findings, Pearson correlation coefficients among individual clinical chemical end points and lipid classes and individual lipid molecular species, Spearman correlation coefficients among individual histological findings and lipid classes and individual lipid molecular species, PE/(PC + PI) mean values of the 4 experimental groups and intergroup comparison with ANOVA, quality parameters of mRNA extraction (260/280 nm (phenol contamination) and the 260/230 ratio values (protein contamination), list of extraction and infusion lipid standards and concentrations, extraction standard recovery, and clinical chemical analysis reagent kit ID numbers, down- and upregulated KEGG pathways and list of genes affected, top 10 down- and upregulated gene ontology pathways with affected gene symbols, Pearson correlations among genes and individual lipid molecular species, above +0.7 and below −0.7 coefficient values, gene ontology pathway enrichment analysis the genes showing the highest positive or correlation with selected individual lipid molecular species (Cer(42:3:2) for positive and PG(38:6) for negative correlations), and listing of individual genes affected (XLSX)

## Abbreviations

FB1: Fumonisin B1
NOAEL: no observed adverse effect level
Sa: sphinganine
So: sphingosine
PC: phosphatidylcholine (diacyl)
PC-O: phosphatidylcholine (alkyl-acyl)
PE: phosphatidylethanolamine (diacyl)
PE-P: phosphatidylethanolamine (alkenyl-acyl)
PI: phosphatidylinositol
PS: phosphatidylserine
PA: phosphatidic acid
PG: phosphatidylglycerol
BMP: bis­(monoacylglycero)­phosphate
CL: cardiolipin
L: lyso derivatives
Cer: ceramide
CerS: ceramide synthase
SM: sphingomyelin
TG: triacylglycerol
DG: diacylglycerol
CE: cholesteryl ester
Chol: cholesterol
MS: mass spectrometry
AST: aspartate aminotransferase
ALT: alanine aminotransferase
LDH: lactate dehydrogenase
CK: creatine kinase
HDL: high density lipoprotein
LDL: low density lipoprotein
GGT: gamma-glutamyl transferase
