# Supplementary material for: Liver Lipidomics, Histology, Transcriptomics, and Clinical Chemistry of Rats Intraperitoneally Treated with Fumonisin B1 for 5 days
Source: J Agric Food Chem. 2025 Sep 17;73(39):24975–96. doi: 10.1021/acs.jafc.5c05715 (PMC12492397; doi:10.1021/acs.jafc.5c05715)
Supplement: Supplementary file 1 [file jf5c05715_si_001.pdf]

supporting information file for the article:

Liver lipidomics, histology, transcriptomics and clinical chemistry of rats intraperitoneally treated with fumonisin B1 for five days

András Szabó *§ - Mária Péter §, Gábor Balogh, Zsolt Török, Zoltán Kóta, László Vígh, Omeralfaroug Ali, Botond Timár, György Kövér, Tibor Nagy, Ferenc Olasz, Brigitta Bóta, Örs Petneházy, Patrik Gömbös, Edward Agyarko, Nguyen Anh Thi, Éva Varga-Visi, Melinda Kovács

* corresponding author; § These authors contributed equally to the manuscript.

Affiliations:

András Szabó - Agribiotechnology and Precision Breeding for Food Security National Laboratory, Department of Physiology and Animal Health, Institute of Physiology and Nutrition, Hungarian University of Agriculture and Life Sciences, Kaposvár, Hungary; HUN-REN-MATE Mycotoxins in the Food Chain Research Group, Kaposvár, Hungary. E-mail address: szabo.andras@uni-mate.hu.

AUTHORS

Mária Péter, Gábor Balogh, Zsolt Török, László Vígh - HUN-REN Biological Research Center, Institute of Biochemistry, Szeged, Hungary.

Zoltán Kóta - Single Cell Omics Advanced Core Facility, Hungarian Centre of Excellence for Molecular Medicine, Szeged, Hungary.

Omeralfaroug Ali, Örs Petneházy, Patrik Gömbös, Edward Agyarko, Éva Varga-Visi - Agribiotechnology and Precision Breeding for Food Security National Laboratory, Department of Physiology and Animal Health, Institute of Physiology and Nutrition, Hungarian University of Agriculture and Life Sciences, Kaposvár, Hungary.

Melinda Kovács - Agribiotechnology and Precision Breeding for Food Security National Laboratory, Department of Physiology and Animal Health, Institute of Physiology and Nutrition, Hungarian University of Agriculture and Life Sciences, Kaposvár, Hungary; HUN-REN-MATE Mycotoxins in the Food Chain Research Group, Kaposvár, Hungary.

Botond Timár - Department of Pathology and Experimental Cancer Research, Semmelweis University, Budapest, Hungary.

György Kövér, Nguyen Anh Thi - Department of Animal Science, Institute of Animal Breeding Sciences, Hungarian University of Agricultural and Life Sciences, Kaposvár, Hungary.

Tibor Nagy, Ferenc Olasz - Agribiotechnology and Precision Breeding for Food Security National Laboratory, Institute of Genetics and Biotechnology, Hungarian University of Agriculture and Life Sciences, Gödöllő, Hungary.

Brigitta Bóta - HUN-REN-MATE Mycotoxins in the Food Chain Research Group, Kaposvár, Hungary.

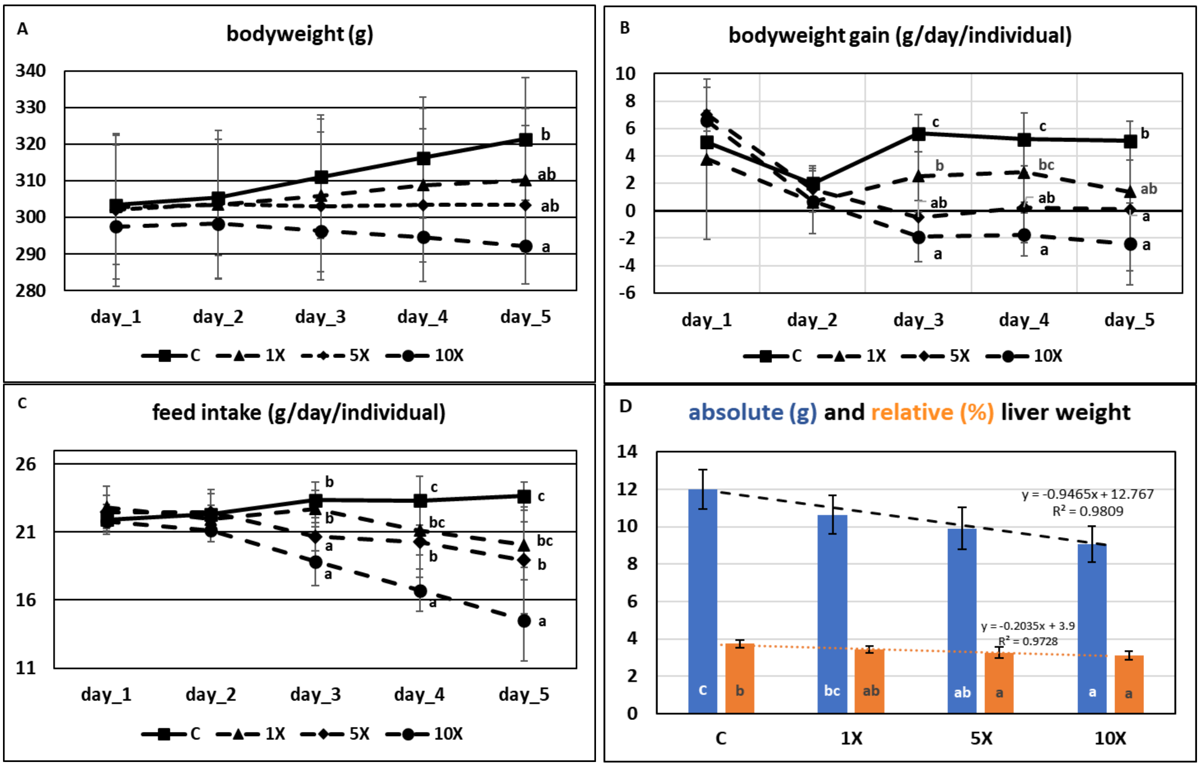

Figure 1: (A) Bodyweight of the rats during the 5 study days; (B) Bodyweight gain during the 5-day period in the 4 groups; (C) Feed intake during the FB1 administration period; (D) absolute and relative liver weight at the end of experiment. (dots and columns represent means±SD) (a,b,c in the plots indicate significant intergroup differences ($p<0.05$) of means at the distinct sampling days if the indices differ).

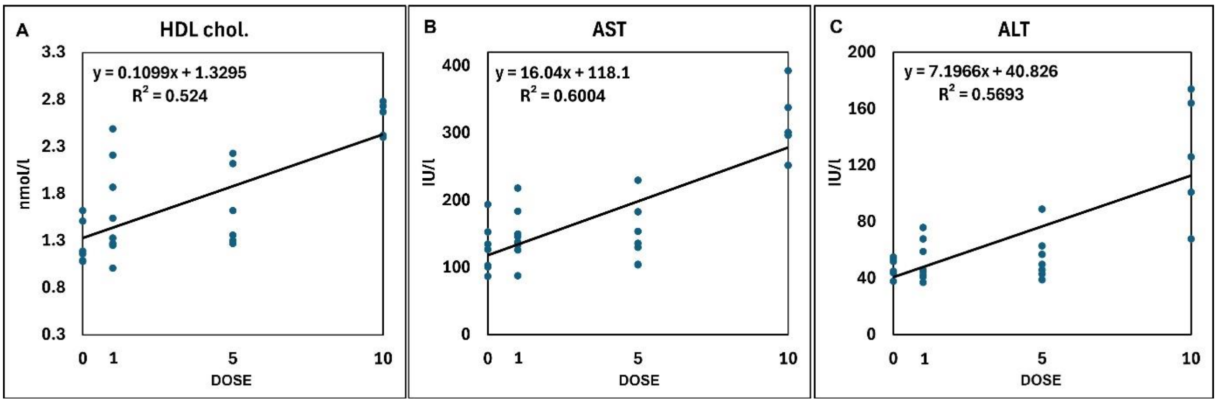

Figure 2. Linear, dose-dependent alterations of the HDL cholesterol (A), AST (B) and ALT (C) activities in the 4 experimental groups after 5 days (dots represent individual values).

Table 1. Mean and range descriptive data of the distinct histological findings logged in the liver samples (means are calculated for n=8/group, *: checked but not discovered, PAS: periodic acid Schiff staining)

group	C			1X			5X			10X		
	mean	min.	max.	mean	min.	max.	mean	min.	max.	mean	min.	max.
**Fatty change**	0.25	0	1	0	0	0	0	0	0	0.25	0	1
**Hydropic degeneration**	0.13	0	1	0.13	0	1	0.75	0	1	1.13	1	2
**Apoptosis/Mitosis/Regenerative sign**	0	0	0	0	0	0	2	1	3	2.75	2	4
**Hepatocellular hypertrophy**	0.38	0	1	0.38	0	1	0.88	0	1	1	1	1
**Necrosis***	0	0	0	0	0	0	0	0	0	0	0	0
**Portal changes**	0	0	0	0	0	0	0.38	0	1	1	1	1
**Loss of PAS positivity**	0.88	0	1	0.75	0	1	2.25	1	4	2.87	2	4
**Total score value**	1.63	0	3	1.25	0	3	6.25	3	10	9	7	12

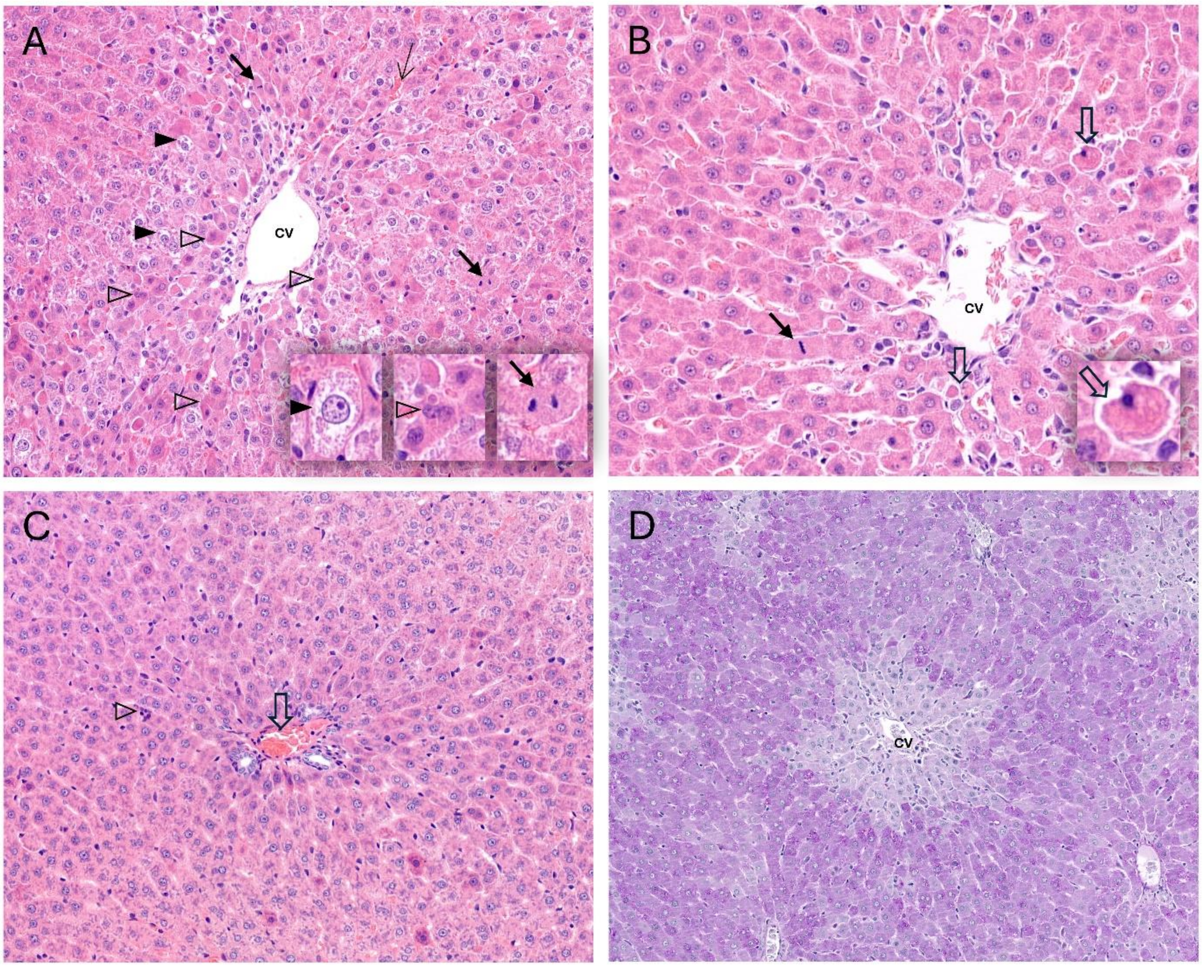

Figure 3. Histologic changes observed in the rat liver at 10X FB1 dose after 5 consecutive days. **(A)** Hydropic change of hepatocytes (filled arrowhead and insert) with pericentral mitotic figures (arrow and insert) and with parenchymal collapse with atrophic hepatocytes (empty arrowhead and insert) (63X, HE staining). **(B)** Presence of apoptosis (Councilman bodies, empty arrowhead, insert) and mitotic figures (arrow) in a pericentral (CV: central vein) predominance (63X, HE staining). **(C)** Minimal portal changes, predominantly congestion (empty arrow) with occasional lymphocytic infiltrates in the periportal region (empty arrowhead) (63X, HE staining). **(D)** Loss of PAS staining intensity (positivity) in the pericentral region (63X, PAS staining).

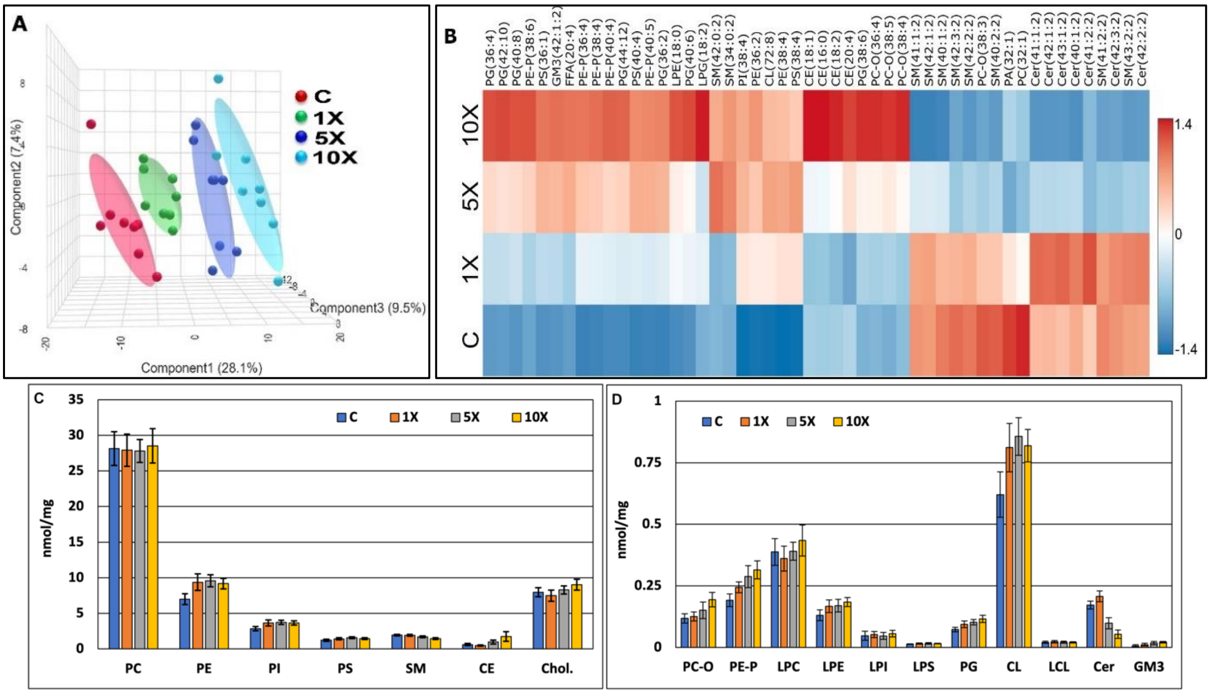

Figure 4. Overview of the lipidomic response to i.p. FB1 treatment. (A) PLS-DA score plot and (B) heatmap representation of hierarchical cluster analysis based on the entire hepatic lipidomics dataset (top 50 most significant species were selected based on ANOVA, Euclidean distance, clustering algorithm Ward; only group averages are shown; heat color code represents normalized values (z-scores)), FB1-induced changes at **lipid class level** in the (C) major and (D) minor lipid classes (bars represent means of 8 ind. values ± SD).

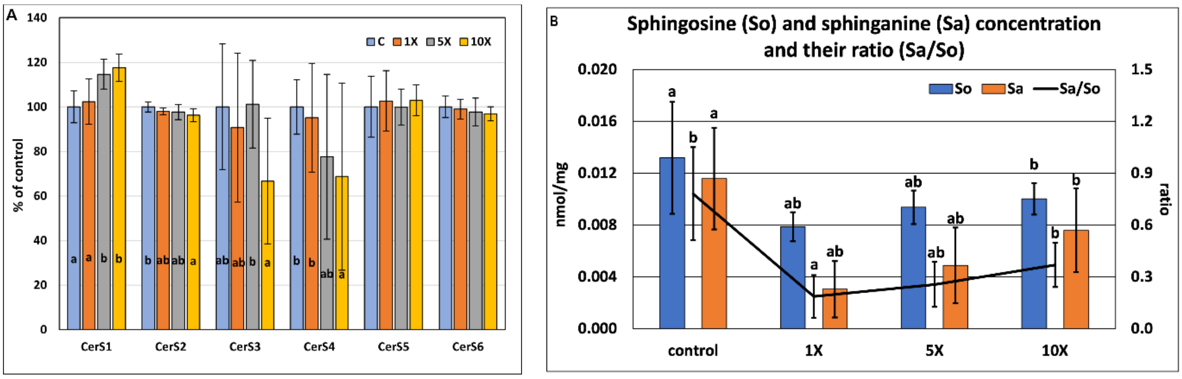

Figure 5. (A) CerS isoforms' relative expression in the rat livers (100% refers to the control). (B) The concentration of hepatic sphinganine (Sa) and sphingosine, and their ratio. (a,b: different indices mark significantly different group means at $p<0.05$ by ANOVA)

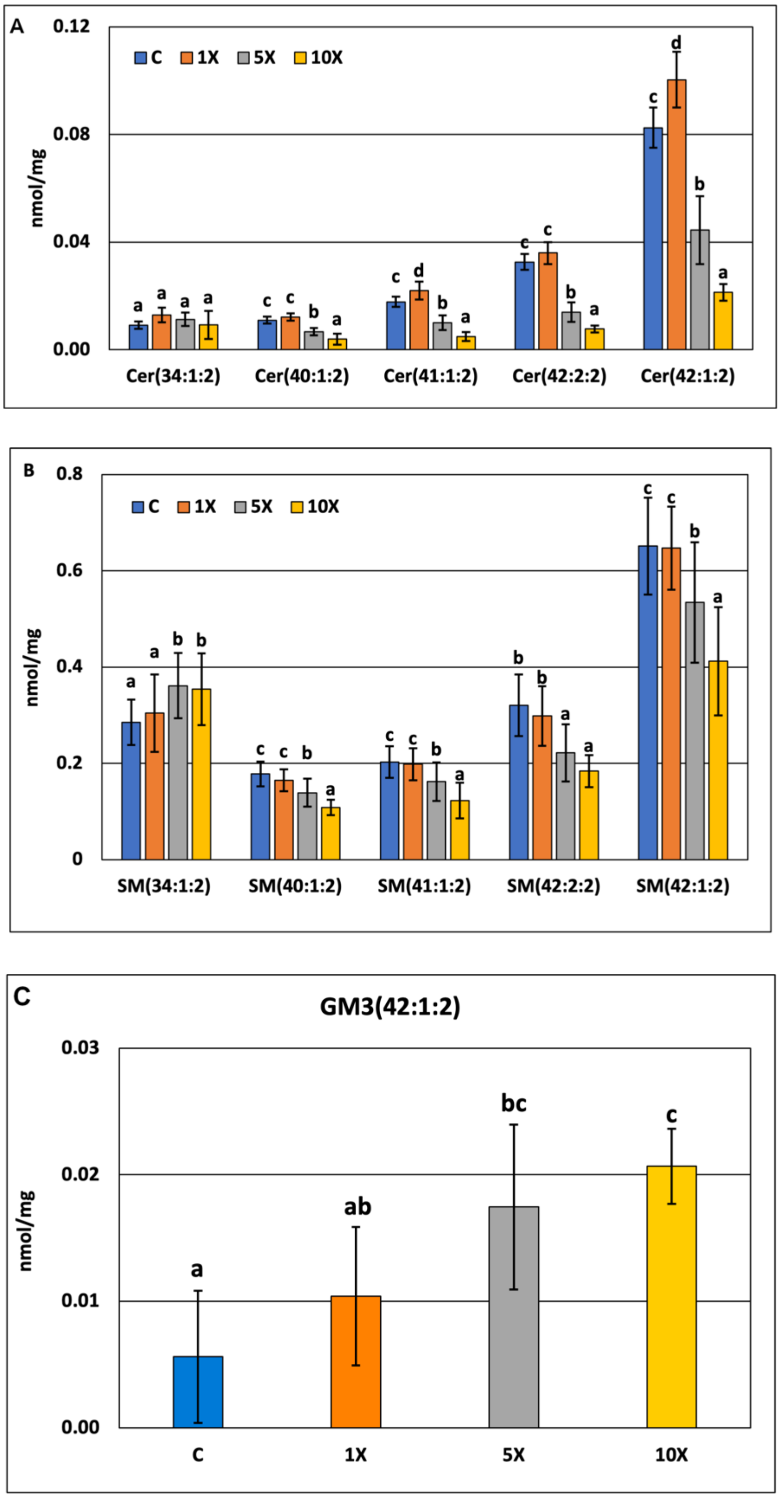

Figure 6. Dose-dependent alterations of some Cer (A), respective SM (B), and the single identified GM3 ganglioside (C) molecular species. (a,b,c: different indices mark significantly different group means at $p<0.05$).

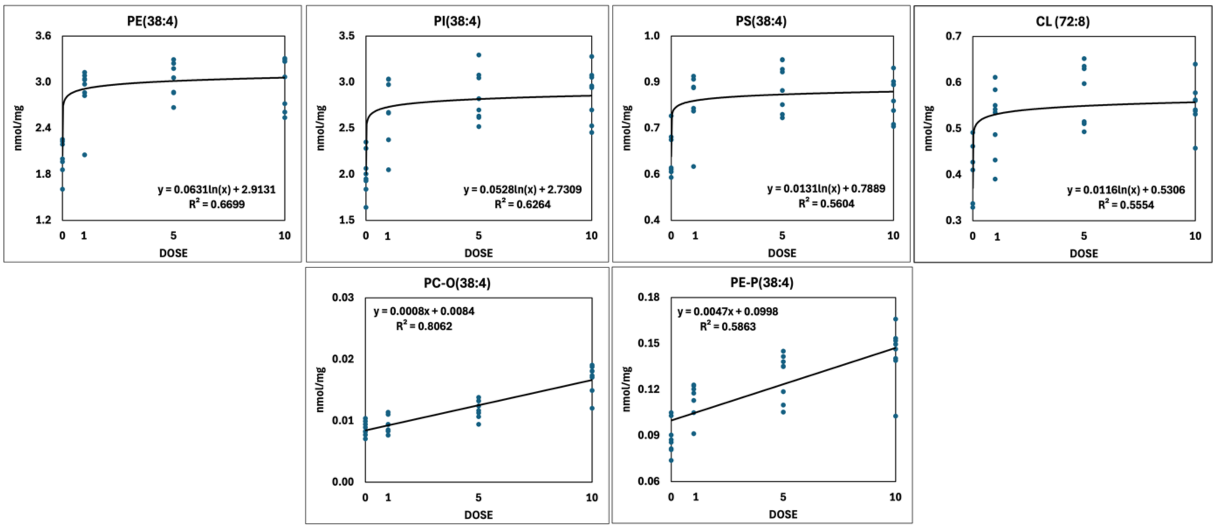

Figure 7. FB1-induced logarithmic and linear dose-response curves of the AA and linoleic acid (CL(72:8) (tetra-linoleoyl CL)) containing lipid molecular species of the glycerophospholipidome (dots represent individual values, n=32).

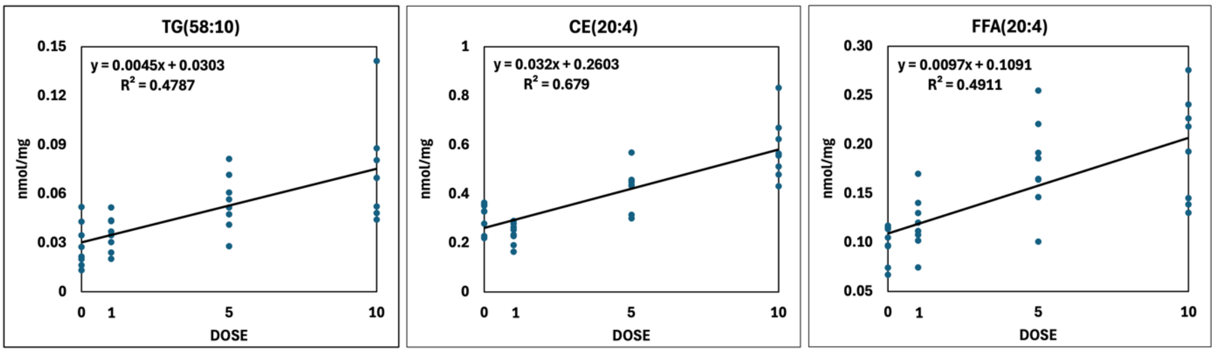

Figure 8. Linear dose-response of AA-containing neutral lipid molecular species in the triglyceride (TG), cholesteryl ester (CE) and free fatty acid (FFA) classes.

Table 2. The Spearman rank correlation coefficients between the identified histological changes and the most abundant lipid molecular species

	PC(38:4)	LPC(18:0)	PE(38:4)	LPE(18:0)	PI(38:4)	LPI(18:0)	PS(38:4)	LPS(20:4)	PG(34:1)	LPG(22:6)	CL(72:8)	LCL(54:6)
**Fatty change**	-0.02	0.34	-0.13	0.05	-0.16	0.09	-0.26	-0.06	0.12	-0.02	-0.11	0.10
**Hydropic degeneration**	0.27	0.56	0.30	0.45	0.35	0.01	0.22	0.13	0.29	0.55	0.31	0.02
**Apoptosis/Mitosis/Regenerative sign**	0.34	***0.66***	0.56	***0.67***	0.53	0.17	0.45	0.26	0.44	***0.67***	0.54	0.11
**Hepatocellular hypertrophy**	0.31	0.29	0.37	0.37	0.37	0.00	0.37	0.37	0.16	0.41	0.39	-0.08
**Portal changes**	0.32	0.59	0.40	***0.61***	0.50	0.15	0.32	0.03	0.43	***0.64***	0.44	0.18
**Loss of PAS positivity**	0.39	***0.74***	0.40	0.57	0.39	0.20	0.28	0.18	0.18	0.53	0.45	0.12
**Total hist. score**	0.41	***0.70***	0.44	0.59	0.43	0.14	0.33	0.23	0.28	0.59	0.47	0.08

	PC-O(38:5)	PE-P(38:4)	SM(42:1:2)	Cer(42:1:2)	GM3(42:1:2)	Sa	So	DG(34:2)	TG(54:2)	CE(20:4)	Chol.	FFA(18:2)
**Fatty change**	0.02	-0.07	0.07	-0.10	-0.10	0.22	0.28	0.38	0.39	0.23	0.21	0.01
**Hydropic degeneration**	***0.69***	0.53	*-0.66*	*-0.82*	***0.68***	0.56	0.36	0.18	-0.21	***0.77***	***0.68***	0.53
**Apoptosis/Mitosis/Regenerative sign**	***0.73***	***0.73***	*-0.85*	*-0.89*	***0.80***	0.40	0.53	0.05	-0.27	***0.82***	***0.61***	***0.70***
**Hepatocellular hypertrophy**	0.42	0.46	-0.48	-0.55	0.57	0.25	0.49	-0.05	-0.18	0.53	0.46	0.45
**Portal changes**	***0.65***	***0.64***	*-0.74*	*-0.77*	***0.64***	0.47	0.57	0.05	-0.18	***0.80***	0.55	***0.63***
**Loss of PAS positivity**	0.57	***0.60***	*-0.66*	*-0.85*	***0.62***	0.52	***0.62***	0.10	-0.28	***0.76***	***0.74***	***0.61***
**Total hist. score**	***0.68***	***0.64***	*-0.71*	*-0.89*	***0.73***	0.52	***0.65***	0.14	-0.19	***0.86***	***0.74***	***0.66***
color scale	-1.00	0.00	1.00									

Values below and over -0.6 and 0.6, respectively, are marked.

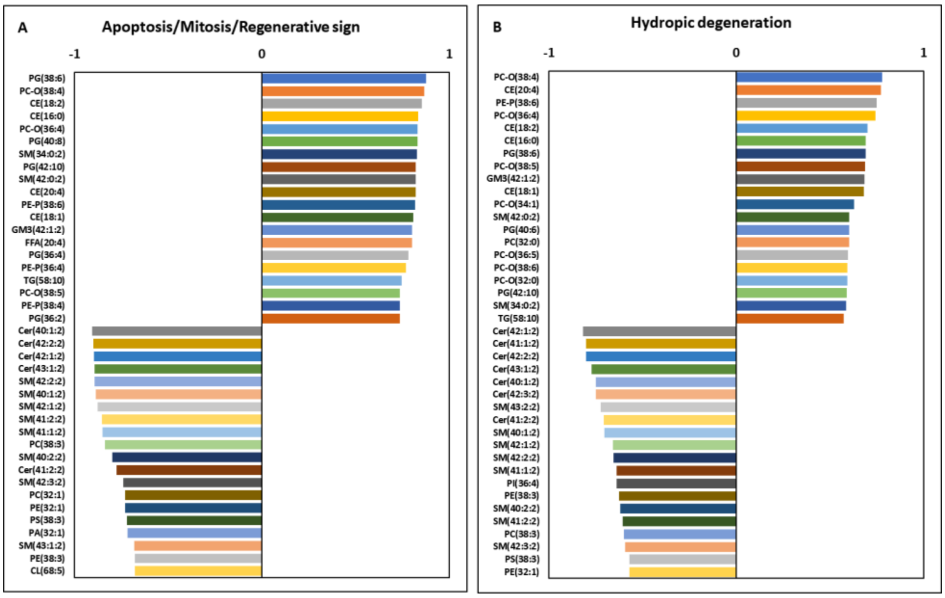

Figure 9. The top 20 strongest positive and negative Spearman correlation coefficients among (A) the apoptosis/mitosis/regenerative signs, and (B) the hydropic degeneration and the individual lipid molecular species.

Table 3. Pearson correlations among the plasma clinical chemical parameters and the most abundant hepatic lipid molecular species

	PC(38:4)	LPC(18:0)	PE(38:4)	LPE(18:0)	PI(38:4)	LPI(18:0)	PS(38:4)	LPS(20:4)	PG(34:1)	LPG(22:6)	CL(72:8)	LCL(54:6)
Total prot.	0.21	0.23	0.49	0.52	0.54	0.24	0.52	0.30	0.33	0.22	0.40	0.25
Albumin	0.12	-0.09	0.40	0.44	0.46	-0.05	0.46	0.19	0.48	0.33	0.36	-0.04
Urea	0.05	0.22	0.33	0.40	0.24	0.19	0.27	0.03	0.25	0.25	0.26	0.05
Uric acid	0.04	0.24	0.07	0.31	0.07	0.07	-0.04	-0.36	0.17	0.36	-0.03	-0.17
Triglyceride	-0.19	-0.33	-0.25	-0.38	-0.30	-0.23	-0.20	0.16	-0.16	-0.39	-0.17	-0.18
Total chol.	0.29	0.54	0.37	0.59	0.48	0.44	0.30	-0.13	0.39	***0.61***	0.25	0.26
HDL chol.	0.34	0.58	0.40	***0.62***	0.51	0.44	0.32	-0.11	0.40	0.58	0.27	0.27
LDL chol.	0.23	0.47	0.33	0.55	0.44	0.41	0.29	-0.15	0.34	0.58	0.25	0.26
LDH	-0.13	-0.09	-0.06	0.06	0.03	-0.17	-0.06	-0.07	0.22	0.03	-0.15	-0.34
AST	0.27	0.39	0.41	0.60	0.52	0.26	0.36	0.03	0.43	0.48	0.32	0.08
ALT	0.28	0.53	0.37	***0.61***	0.46	0.32	0.30	0.00	0.39	0.55	0.32	0.12
GGT	0.10	0.05	0.28	0.38	0.14	-0.13	0.19	-0.14	0.17	0.26	0.14	-0.26
cholinesterase	-0.02	-0.03	-0.35	-0.31	-0.47	-0.30	-0.39	-0.26	-0.21	-0.17	-0.40	-0.30
lipase	0.28	0.40	0.35	0.37	0.41	0.27	0.30	-0.08	0.26	0.27	0.30	0.21
ALP	0.01	-0.14	0.04	-0.03	0.09	-0.11	0.12	0.38	0.12	0.15	0.16	0.01
CK	-0.28	-0.27	0.01	0.07	0.03	-0.16	-0.03	0.04	0.29	-0.03	-0.11	-0.28

	PC-O(38:5)	PE-P(38:4)	SM(42:1:2)	Cer(42:1:2)	GM3(42:1:2)	Sa	So	DG(34:2)	TG(54:2)	CE(20:4)	Chol.	FFA(18:2)
Total prot.	0.24	***0.62***	-0.44	-0.37	0.39	0.26	0.38	-0.10	-0.23	0.40	0.20	0.45
Albumin	0.32	***0.63***	-0.55	-0.32	0.38	0.12	0.25	-0.27	-0.22	0.32	0.05	0.40
Urea	0.31	0.39	-0.32	-0.26	0.29	0.18	0.10	-0.11	-0.08	0.51	0.01	0.09
Uric acid	0.31	0.28	-0.46	-0.35	0.16	0.35	0.35	-0.16	0.05	0.52	0.17	0.03
Triglyceride	-0.25	-0.39	0.35	0.28	-0.28	-0.34	-0.25	0.02	0.21	-0.24	-0.29	-0.27
Total chol.	0.48	***0.62***	***-0.62***	-0.56	0.47	0.50	0.44	-0.10	-0.19	0.59	0.46	0.42
HDL chol.	0.50	***0.64***	***-0.62***	-0.57	0.49	0.48	0.46	-0.07	-0.18	***0.62***	0.47	0.44
LDL chol.	0.40	0.57	-0.56	-0.48	0.42	0.50	0.38	-0.10	-0.23	0.48	0.42	0.39
LDH	-0.02	0.14	-0.35	-0.29	0.05	0.16	0.26	-0.29	-0.13	0.20	0.10	-0.03
AST	0.52	***0.66***	***-0.73***	***-0.62***	0.54	0.42	0.46	-0.27	-0.17	***0.68***	0.46	0.36
ALT	0.56	0.60	***-0.69***	***-0.63***	0.49	0.45	0.34	-0.20	-0.16	***0.72***	0.58	0.29
GGT	0.32	0.36	-0.47	-0.46	0.30	0.26	0.16	-0.36	-0.39	0.37	0.09	0.17
cholinesterase	-0.18	-0.34	0.30	0.12	-0.22	0.16	0.32	0.20	0.29	-0.03	-0.16	0.03
lipase	0.23	0.41	-0.32	-0.23	0.43	0.04	0.27	-0.25	-0.21	0.39	0.10	0.16
ALP	0.14	0.02	0.00	0.11	0.25	-0.14	-0.01	-0.09	-0.04	-0.13	0.11	-0.11
CK	-0.11	0.10	-0.26	-0.11	-0.01	-0.02	0.22	-0.27	-0.10	-0.01	-0.04	-0.04
color scale	-1	0	1									

Values below and over -0.6 and 0.6, respectively are marked.

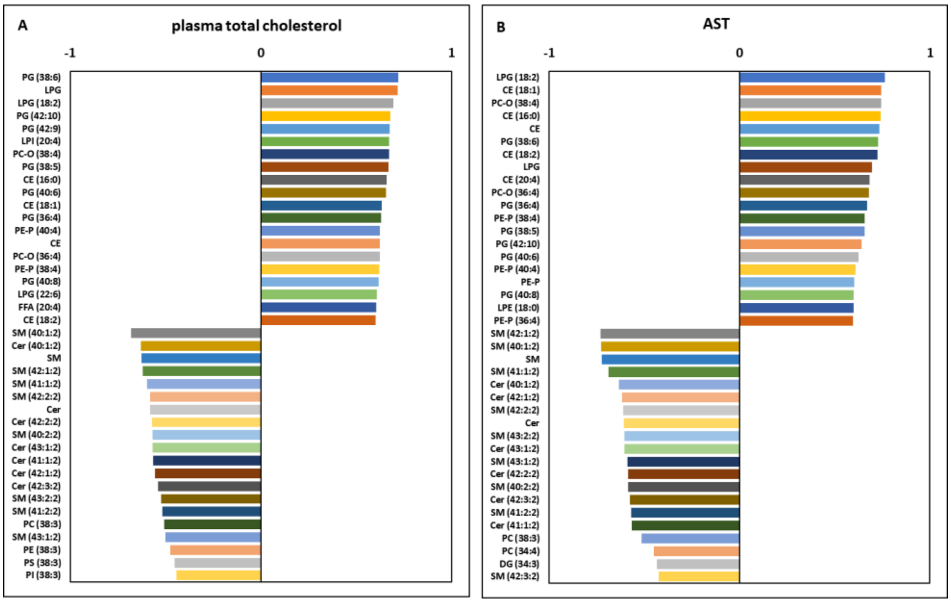

Figure 10. The top 20 positively and negatively correlating lipid molecular species' Pearson coefficient values for (A) plasma total cholesterol and (B) AST activity.

Table 4. The top 20 down- and upregulated genes in the liver (beta is the slope of the regression model set as beta vs. FB1 dose, fitting was performed on 4x8 individual data)

Rank order	DOWNREGULATED	Beta	UPREGULATED	Beta
**1**	LOC103693974	-0.43	**Mmp12**	0.65
**2**	Lgsn	-0.35	LOC102549755	0.53
**3**	Stac3	-0.31	Abcb1b	0.51
**4**	**Pla2g2a**	-0.31	**Trem2**	0.50
**5**	LOC102547849	-0.29	Cyp2c24	0.48
**6**	LOC102551428	-0.28	Atf3	0.46
**7**	LOC100910106	-0.28	LOC103693477	0.42
**8**	Inmt	-0.28	Mmp3	0.41
**9**	LOC102553657	-0.28	**Ccl2**	0.41
**10**	**Pnpla3**	-0.27	Tex36	0.40
**11**	Clic3	-0.27	Gpnmb	0.39
**12**	LOC102553540	-0.26	LOC102555660	0.39
**13**	LOC102551365	-0.26	Bcat1	0.39
**14**	LOC103690303	-0.25	Maff	0.37
**15**	Cyp26a1	-0.24	Spp1	0.37
**16**	LOC102547310	-0.24	Neurl3	0.36
**17**	Papss1	-0.23	**Cxcl10**	0.36
**18**	Prss55	-0.23	**Pla2g2d**	0.35
**19**	Per2	-0.22	Ccl1	0.35
**20**	Olr1305	-0.22	Sfn	0.34

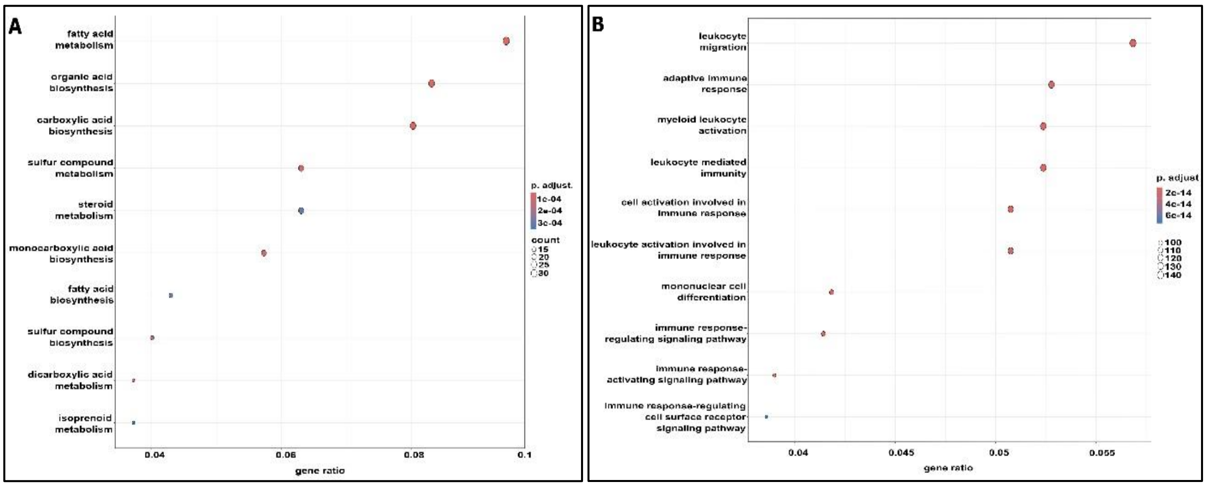

Figure 11. The top 10 most important biochemical processes downregulated (A) or upregulated (B) in the rat livers by FB1.

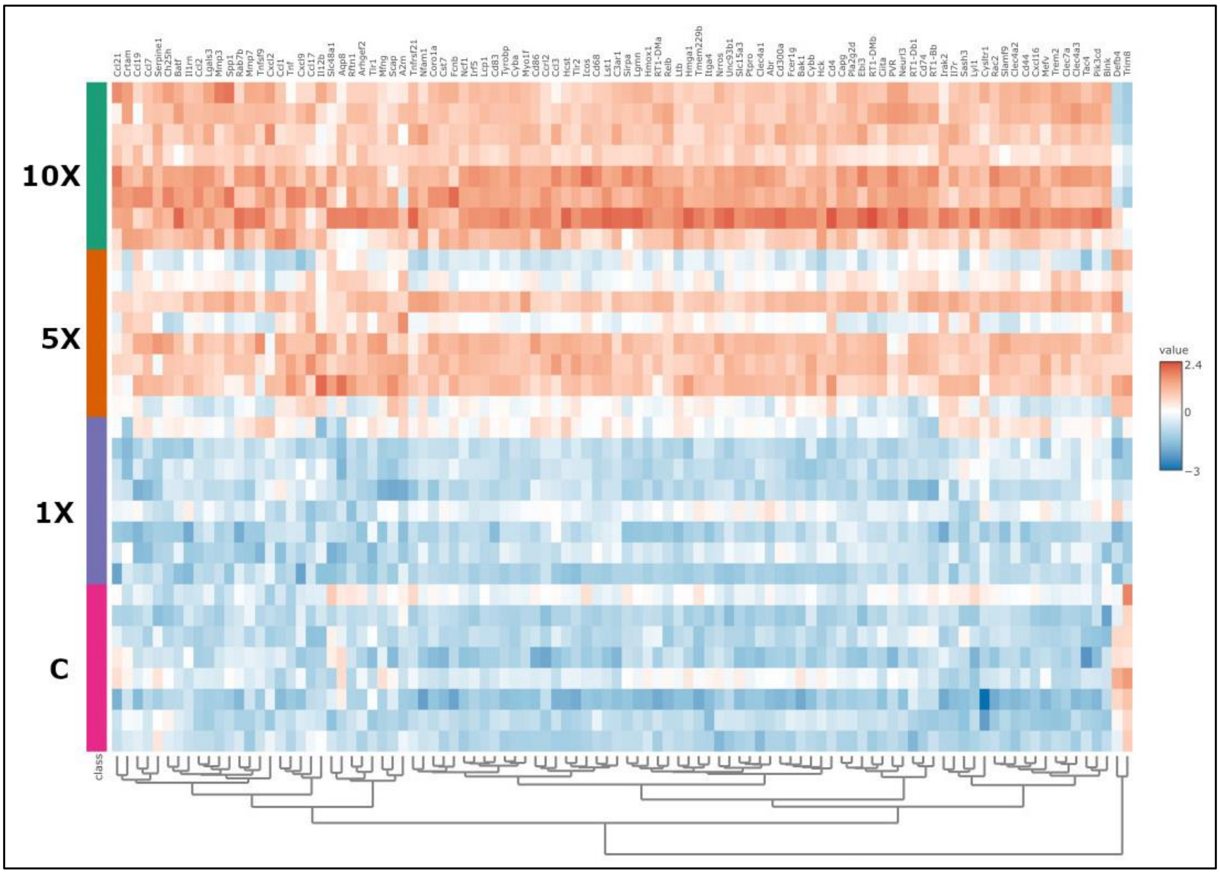

Figure 12. Hierarchical clustering based on the top 100 immunologically competent genes.

Table 5. Top 20 positive and negative correlations among lipid molecular species and individual genes

Rank order	Lipid species	Gene	Corr. coeff.	Lipid species	Gene	Corr. coeff.
**1**	**Cer(42:3:2)**	Mmp12	-0.9523	**PG(38:6)**	LOC102549755	0.9226
**2**	Cer(42:2:2)	Mmp12	-0.9422	**PG(38:6)**	LOC103693477	0.9200
**3**	SM(40:1:2)	Pla2g2d	-0.9281	**PG(38:6)**	Pla2g7	0.9043
**4**	**Cer(42:3:2)**	Slc2a6	-0.9252	PC-O(38:4)	LOC102549755	0.9041
**5**	**Cer(42:3:2)**	Irf5	-0.9237	PG(40:8)	C3ar1	0.8992
**6**	Cer(42:2:2)	Relb	-0.9226	**PG(38:6)**	Cyp2c24	0.8946
**7**	SM(42:1:2)	Pla2g2d	-0.9225	**PG(38:6)**	Gpnmb	0.8946
**8**	**Cer(42:3:2)**	Cd83	-0.9191	**PG(38:6)**	Abcb1b	0.8945
**9**	Cer(41:1:2)	Cd5l	-0.9189	**PG(38:6)**	Lgals3	0.8912
**10**	**Cer(42:3:2)**	Relb	-0.9175	**PG(38:6)**	Iglon5	0.8902
**11**	Cer(41:1:2)	Mcm2	-0.9162	PG(40:8)	Cyp2c24	0.8893
**12**	Cer(42:1:2)	Cd5l	-0.9152	**PG(38:6)**	Clec4a3	0.8879
**13**	sum_Cer	RT1-Bb	-0.9144	PC-O(38:4)	LOC103693477	0.8837
**14**	Cer(42:2:2)	Cd83	-0.9143	PC-O(38:4)	Gpnmb	0.8831
**15**	Cer(42:1:2)	Mmp12	-0.9138	PC-O(36:4)	Gpnmb	0.8827
**16**	SM(42:1:2)	Capg	-0.9122	**PG(38:6)**	Clec7a	0.8818
**17**	sum_Cer	Cd5l	-0.9120	PC-O(36:4)	LOC103693477	0.8806
**18**	**Cer(42:3:2)**	Bcat1	-0.9117	PC-O(38:4)	Neurl3	0.8792
**19**	**Cer(42:3:2)**	Cd5l	-0.9105	**PG(38:6)**	Ebi3	0.8779
**20**	SM(40:1:2)	Capg	-0.9102	**PG(38:6)**	Neurl3	0.8778

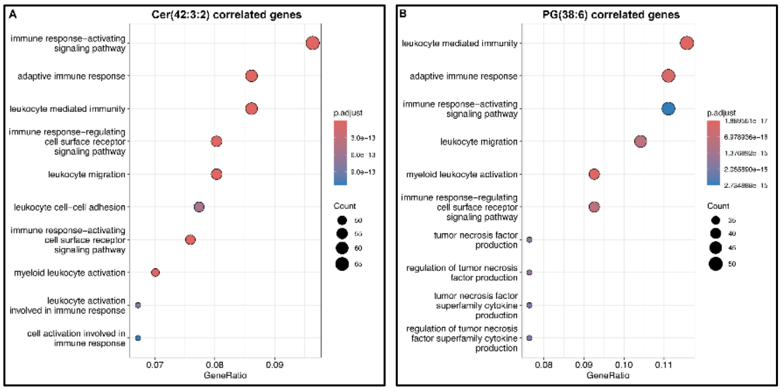

Figure 13. The top 10 biochemical processes regulated by genes in strong correlation with Cer(42:3:2) (A) and PG(38:6) (B), as assessed with GO analysis.

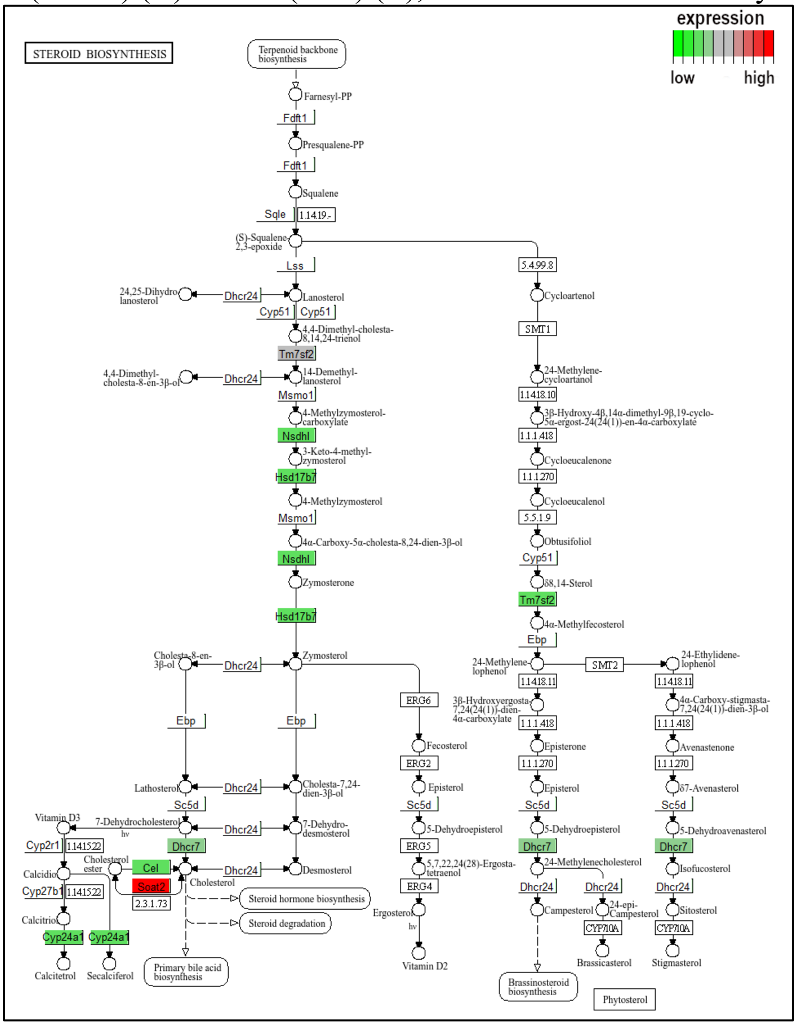

Figure 14. Modulation of the steroid biosynthesis (control vs. 10X level FB1 treatment, mapped with Pathview on a KEGG pathway).

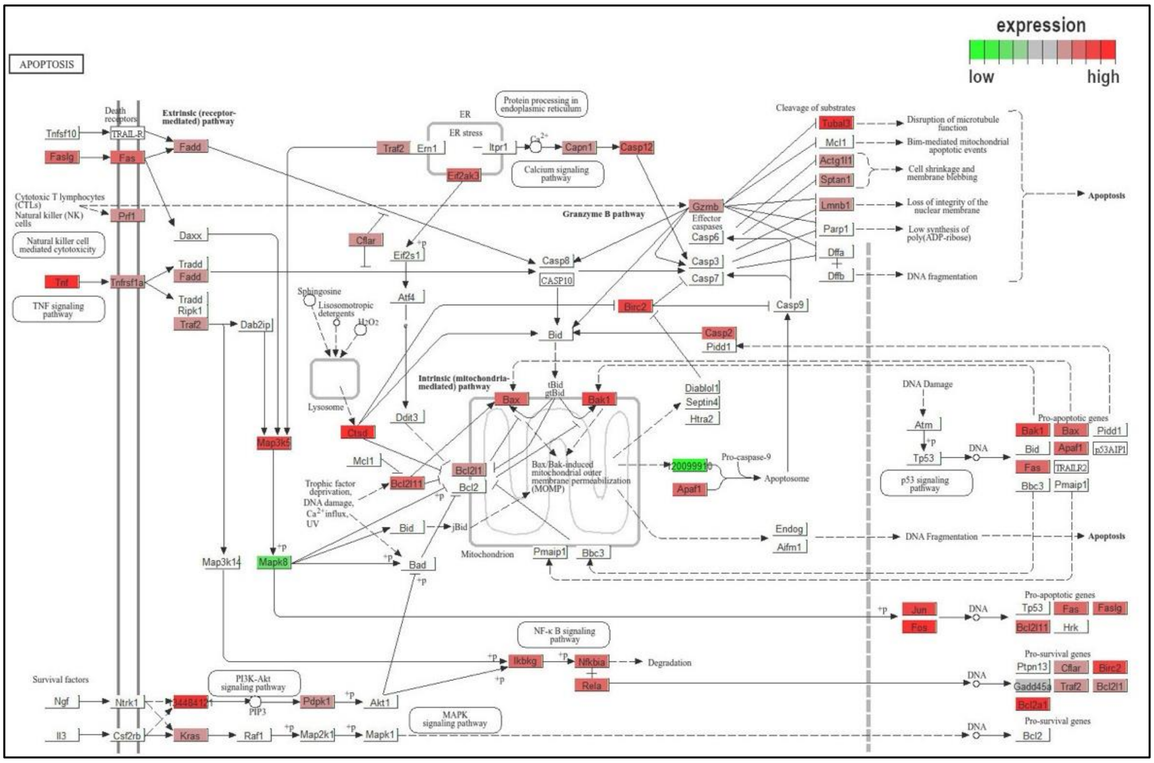

Figure 15. The FB1-induced modification of the regulation of apoptosis in the rat liver. (control vs. 10X FB1 treatment, mapped with Pathview on a KEGG pathway).
